# Advancing patient-centered cancer care: a systematic review of electronic patient-reported outcome measures

**DOI:** 10.3389/fresc.2024.1427712

**Published:** 2024-09-25

**Authors:** Hosna Salmani, Somayeh Nasiri, Mahdi Alemrajabi, Maryam Ahmadi

**Affiliations:** ^1^ Department of Health Information Management, School of Health Management and Information Sciences, Iran University of Medical Sciences, Tehran, Iran; ^2^ Gastrointestinal and Liver Diseases Research Center, Iran University of Medical Sciences, Tehran, Iran

**Keywords:** electronic patient-reported outcome measures, ePROMs, cancer care, measurement instruments, patient assessment

## Abstract

**Background:**

Electronic Patient-Reported Outcome Measures (ePROMs) have emerged as valuable tools in cancer care, facilitating the comprehensive assessment of patients’ physical, psychological, and social well-being. This study synthesizes literature on the utilization of ePROMs in oncology, highlighting the diverse array of measurement instruments and questionnaires employed in cancer patient assessments. By comprehensively analyzing existing research, this study provides insights into the landscape of ePROMs, informs future research directions, and aims to optimize patient-centred oncology care through the strategic integration of ePROMs into clinical practice.

**Methods:**

A systematic review was conducted by searching peer-reviewed articles published in academic journals without time limitations up to 2024. The search was performed across multiple electronic databases, including PubMed, Scopus, and Web of Science, using predefined search terms related to cancer, measurement instruments, and patient assessment. The selected articles underwent a rigorous quality assessment using the Mixed Methods Appraisal Tool (MMAT).

**Results:**

The review of 85 studies revealed a diverse range of measurement instruments and questionnaires utilized in cancer patient assessments. Prominent instruments such as the European Organisation for Research and Treatment of Cancer Quality of Life Questionnaire Core 30 (EORTC QLQ-C30) and the Patient Reported Outcome-Common Terminology Criteria for Adverse Events (PRO-CTCAE) were frequently referenced across multiple studies. Additionally, other instruments identified included generic health-related quality of life measures and disease-specific assessments tailored to particular cancer types. The findings indicated the importance of utilizing a variety of measurement tools to comprehensively assess the multifaceted needs and experiences of cancer patients.

**Conclusion:**

Our systematic review provides a comprehensive examination of the varied tools and ePROMs employed in cancer care, accentuating the perpetual requirement for development and validation. Prominent instruments like the EORTC QLQ-C30 and PRO-CTCAE are underscored, emphasizing the necessity for a thorough assessment to meet the multifaceted needs of patients. Looking ahead, scholarly endeavours should prioritize the enhancement of existing tools and the creation of novel measures to adeptly address the evolving demands of cancer patients across heterogeneous settings and populations.

## Introduction

Cancer care involves a complex and multifaceted approach, encompassing diagnosis, treatment, and supportive care interventions aimed at enhancing patient outcomes and quality of life. Within the landscape of cancer care, the selection and application of appropriate assessment tools are pivotal in ensuring the accuracy, reliability, and validity of patient symptom evaluations. In oncology, effectively managing symptoms related to both the disease and treatment toxicity is paramount for improving patients’ quality of life (QoL). However, under-detection and under-reporting of symptoms can hinder optimal supportive care delivery. Symptom monitoring through Patient-reported outcomes (PROs) offers an evidence-based solution to bridge the gap between clinician recognition and patient self-reporting (1). PROs offer an evidence-based solution to bridge the gap between clinician recognition and patient self-reporting, allowing for a comprehensive assessment of physical, psychological, and social well-being. PROs play a crucial role in modern oncology care, allowing patients to directly report on their health status without interpretation by clinicians (2, 3).

The emergence of electronic Patient-Reported Outcome Measures (ePROMs) represents a promising advancement, with digital solutions showing significant benefits in improving satisfaction, treatment adherence, symptom control, and overall clinical outcomes (2–4). Interest in integrating ePROMs into regular cancer care has grown, driven by a desire to enhance health-related quality of life (HRQOL) and other patient-centred outcomes (5). Dedicated tools known as Patient-Reported Outcome Measures (PROMs), such as questionnaires or standardized interview schedules, facilitate the collection of PROs and promote communication between patients and clinicians (1). These tools serve as invaluable instruments in capturing the complex interplay of disease burden, treatment efficacy, and patient-reported experiences, thereby informing tailored interventions and optimizing the delivery of patient-centred care (6, 7).

ePROM questionnaires provide standardized instruments for eliciting patient-reported information across diverse domains, such as symptomatology, functional status, and treatment satisfaction, thereby supplying clinicians and researchers with structured data to inform clinical decision-making and research endeavours. They can facilitate data collection, management, and analysis, streamlining the process of patient assessment and enabling real-time monitoring of symptoms and treatment outcomes (8) ePROMs have been utilized in questionnaires to gather information from patients, who consistently report that ePROMs are easy to comprehend, timely to fill out, and enhance communication with their oncology team. Clinicians also find that ePROMs help communication with patients, increase patient engagement in consultations, and alter clinical decision-making (5, 9).

Recent studies highlight the positive impact of ePROM interventions, offering features like remote symptom reporting and real-time clinician feedback (5). Moreover, ePROMs have shown promising benefits in improving communication, symptom control, prolonging survival, and reducing hospital admissions and emergency department visits (10). A systematic review by Warnecke et al. found that ePROMs improve the assessment of underrated physical and psychological symptom burdens among oncological patients, highlighting their potential to enhance patient-centred care. However, further research is needed to fully understand their clinical utility and address the challenges associated with their implementation (11, 12).

ePROMs have the potential to significantly enhance patient-centred cancer care by improving symptom assessment, communication, and patient engagement. The use of electronic platforms and mobile technologies enables remote data collection, enhancing accessibility, convenience, and patient engagement while minimizing logistical barriers associated with traditional paper-based assessments. Addressing these challenges requires a comprehensive approach that encompasses technological Despite the evident advantages of ePROMs, their widespread adoption and implementation present notable challenges, such as technological barriers, health literacy disparities, and concerns regarding data security, privacy, and confidentiality innovation, healthcare policy reform, and patient education initiatives to improve the equitable and ethical integration of assessment tools in cancer care. This systematic review aims to provide a comprehensive analysis of the landscape of assessment tools, questionnaires, and ePROMs utilized in cancer care, informing future research directions, clinical practice guidelines, and policy initiatives aimed at optimizing patient-centred oncology care.

To guide this analysis, the research questions for our systematic review are formulated as follows:

  RQ1. What are the existing ePROMs utilized in cancer care?

  RQ2. What are the key characteristics and functionalities of ePROMs used in cancer care, and how do they vary across different measurement tools?

These research questions will facilitate a systematic evaluation of the effectiveness of ePROMs in cancer care and help identify optimal strategies for their successful implementation and broader adoption.

## Methods

### Study design

We conducted a systematic review and addressed all research articles focusing on the utilization of ePROM and their potential for patient-centred solutions in cancer care. The final report follows the Preferred Reporting Items for Systematic Reviews and Meta-Analyses (PRISMA) guidelines for reporting systematic reviews. Our study encompasses several key steps including search strategy, inclusion and exclusion criteria, study selection, quality appraisal, and data extraction and synthesis to ensure a comprehensive and rigorous analysis of the available literature (13).

### Search strategy

We searched for articles published in electronic databases up to 2024, using three databases: Scopus, Web of Science and PubMed review. The searches used the following keywords and medical subject heading (MeSH) terms in various combinations. We derived two broad themes that were then combined with the Boolean operators “AND” and “OR”. The first theme in Mesh “electronic Patient Reported Outcomes” was created by the Boolean operator “OR” to combine text words (“electronic Patient-Self Reporting”, OR “electronic Patient Reported Measures”). The second theme “Cancer” was the broad aspect created for the search strategy. Additionally, a backward snowball search will be employed to ensure comprehensive coverage of relevant articles (See Appendix Table A1).

### Inclusion and exclusion criteria

We included papers based on eligibility criteria with the following characteristics: (1) published in English, (2) papers related to electronic patient-reported outcomes, electronic patient-reported measures, and electronic self-reporting (3) articles with various research types like quantitative, qualitative, and mixed methods, (4) Studies assessing quality of life, symptoms, psychological well-being, treatment satisfaction, or other relevant outcomes in cancer patients.

We excluded studies that were inaccessible in full text, studies exclusively focused on technical infrastructure, and those emphasizing paper-based or manual Patient Reported Outcome (PRO) versions, books, protocols, standards, framework and guidelines, conference proceedings, dissertations, conference abstracts, reviews, short reports, posters, newspapers, editorials and commentary. Furthermore, unrelated subjects were excluded such as feasibility, paper vs. electronic systems, terminology criteria, clinical alerts, health equity, perspective, experiences, and perception, data and machine learning, models, associations, not related and not cancer, editorial, biology, telemedicine, ethical principles, wearables, gamification, system design and technical innovations, oncology informatics, precision oncology, validity and reliability, economic. We also excluded studies lacking indicators or outcomes for cancer, not using the system as the intervention tool.

### Study selection

Three investigators independently reviewed papers based on titles and abstracts in alignment with the inclusion and exclusion criteria. Irrelevant studies were removed at this stage. One reviewer (HS) conducted the data extraction, while two other reviewers (MA, SN) rechecked the accuracy of the results. All researchers then read and reviewed the full texts to make final inclusion decisions.

### Quality appraisal

The selected articles underwent a rigorous quality assessment using the Mixed Methods Appraisal Tool (MMAT), comprising components for qualitative, quantitative (clinical trials), quantitative (non-clinical trials), descriptive, and mixed methods, incorporating 25 questions. Each affirmative response contributes to a 25% score. Articles surpassing the average in the number of positive responses or fully specified items are categorized as high quality. Those with positive responses ranging from 25% to 50% are classified as medium quality, while those falling below 25% are considered low quality. Following full-text screening and quality appraisal, a total of 85 articles met the inclusion criteria.

### Data extraction and synthesis

An initial data extraction form was developed at this stage of the review. Data elements were extracted from each article that was organized into two sections: general items (author, year, country/state, objective, and participants) and specific items (system tools & metrics, cancer type and type of treatment). The selected papers were summarized in the final step of our methodology and important factors were identified. Thus, the statistical results of systematic reviews were described for outcomes reported in the studies. Subsequently, data extracted from these pertinent articles will undergo thorough narrative analysis and be presented in organized tables and diagrams (See Appendix Table A2).

## Result

**Figure 1:**
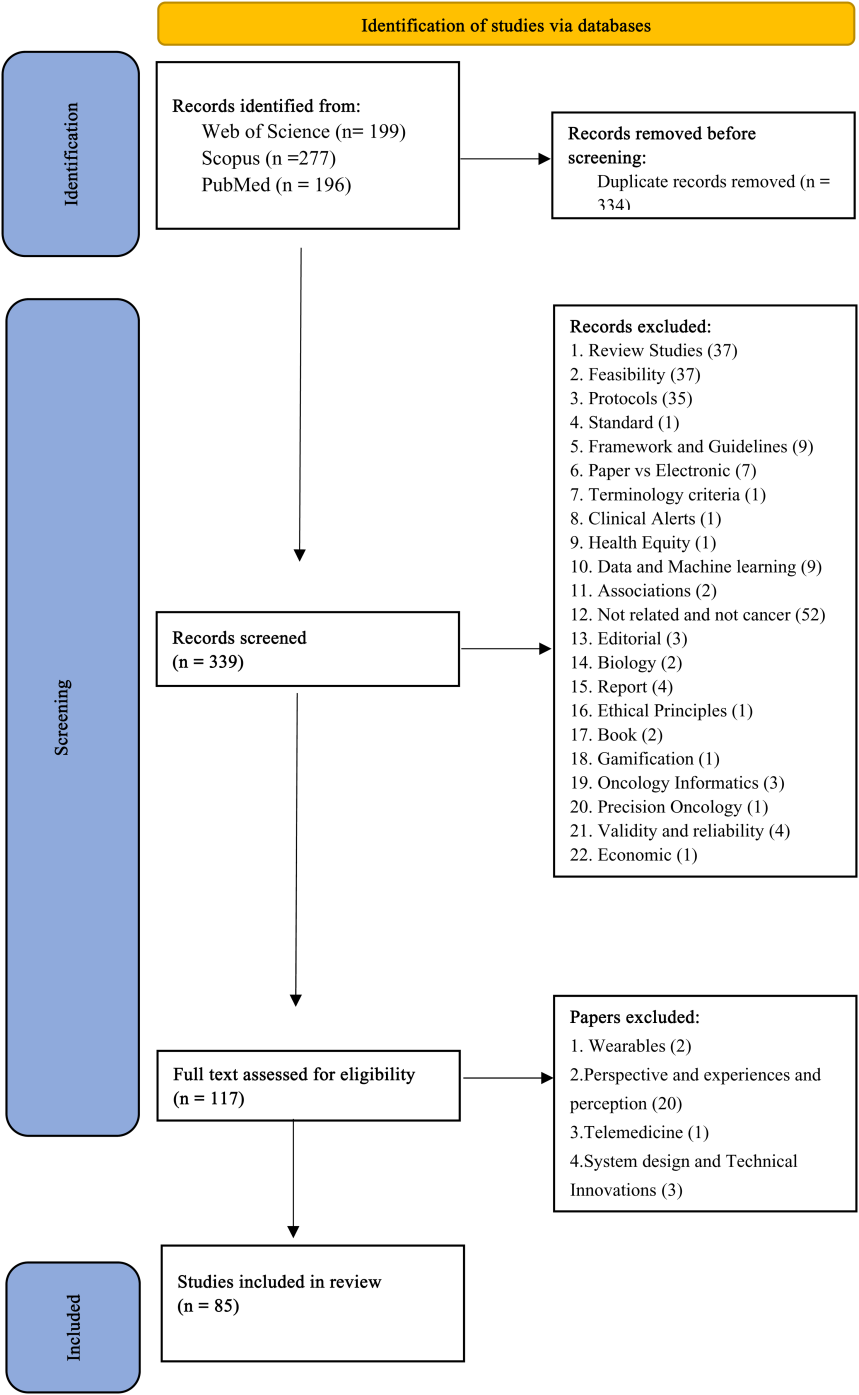
PRISMA flow diagram for selected studies.

In our systematic review, we identified 672 papers, out of which 85 academic papers were included in our systematic review, providing a comprehensive exploration of electronic patient-reported outcome measures for cancer care. We present the key findings regarding the characteristics of the included studies, measurements, and their use as revealed in our systematic review (See Figure 1).

### Characteristics of included studies

**Figure 2:**
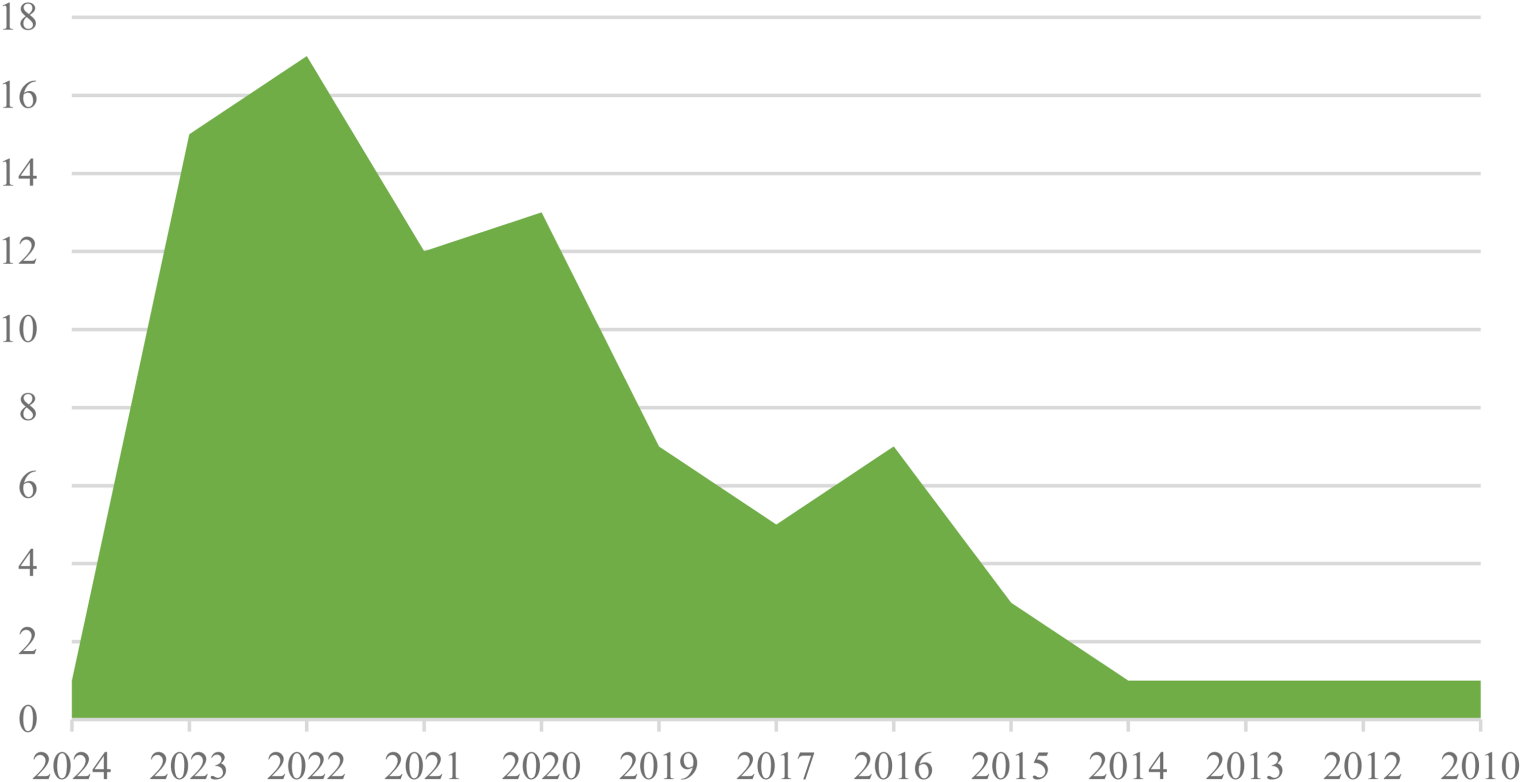
Frequency distribution of articles about ePROM in cancer by year.

The distribution of articles about electronic patient-reported outcome measures in cancer by year indicates that the majority of publications were from the years 2022 and 2023, with 17 and 15 contributions, respectively. Additionally, there were 12 publications from 2021, 13 from 2020, 7 from 2019, 7 from 2016, 5 from 2017, 3 from 2015, and 1 publication each from 2024, 2014, 2013, 2012, and 2010 (See Figure 2).

**Figure 3:**
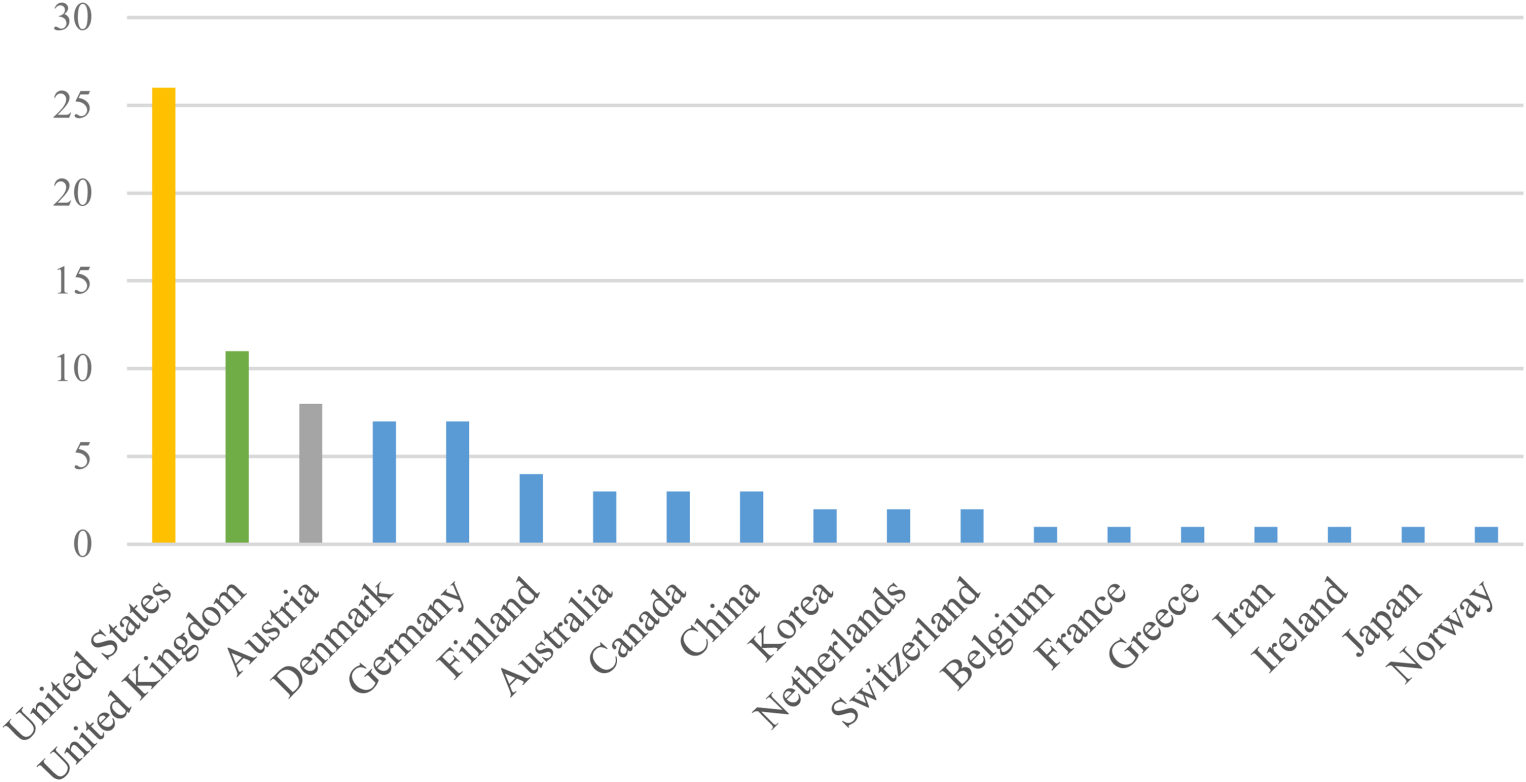
Frequency distribution of articles about ePROM in cancer by country.

According to Figure 3, the United States emerged as the leading source of publications, followed by the United Kingdom in second place and Austria in third. Additionally, Belgium, France, Greece, Iran, Ireland, Japan, and Norway each made a single contribution.

### Type of cancers

**Figure 4:**
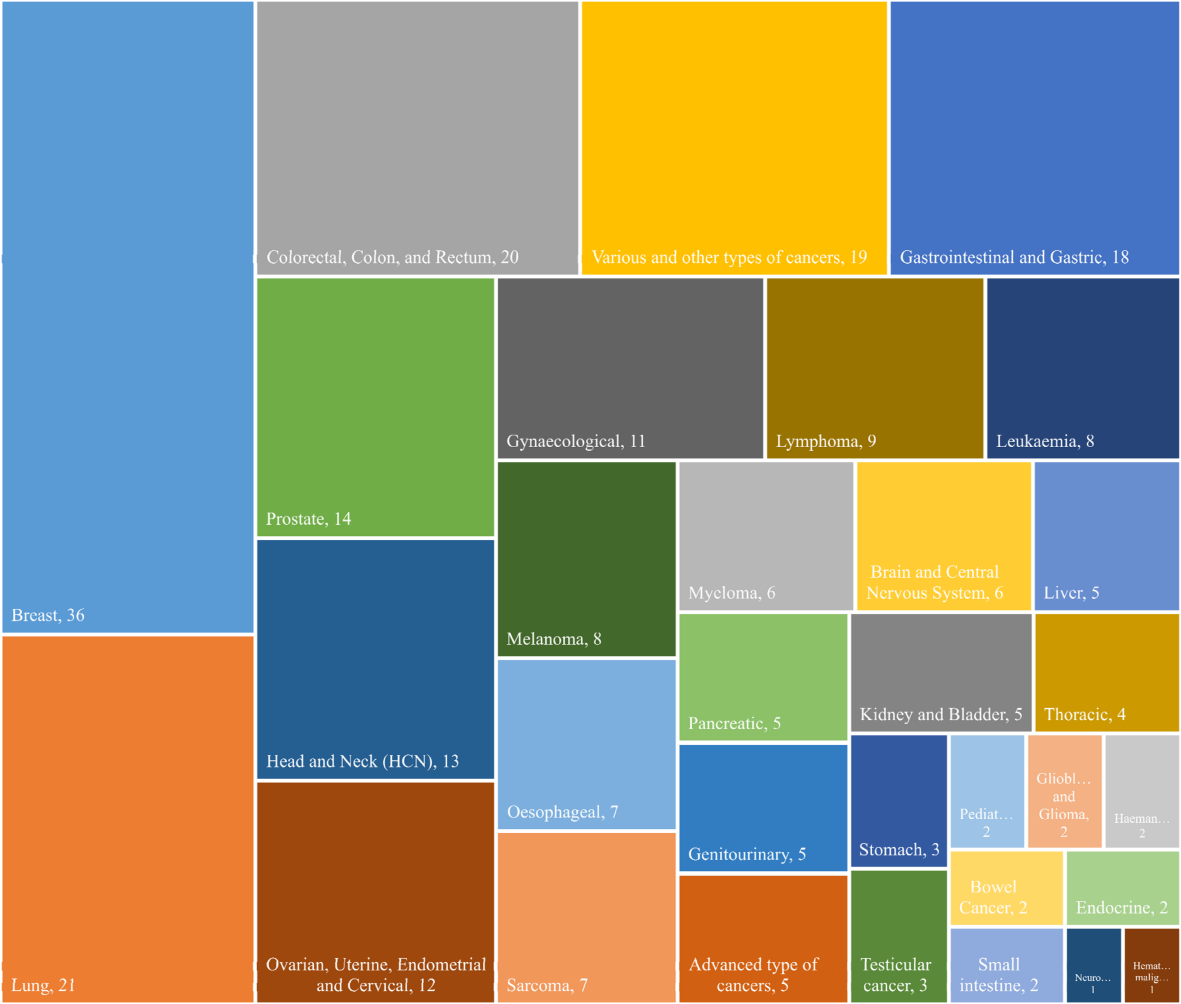
Frequency distribution of studies articles about ePROM according to cancer types.

The type and frequency of cancer within the ePROM study are indicated in Figure 4. This figure highlights the prevalence of various malignancies across the studies. A total of 32 different types of cancer were identified, with breast cancer emerging as the most frequently studied.

### Type of cancer treatments

**Figure 5:**
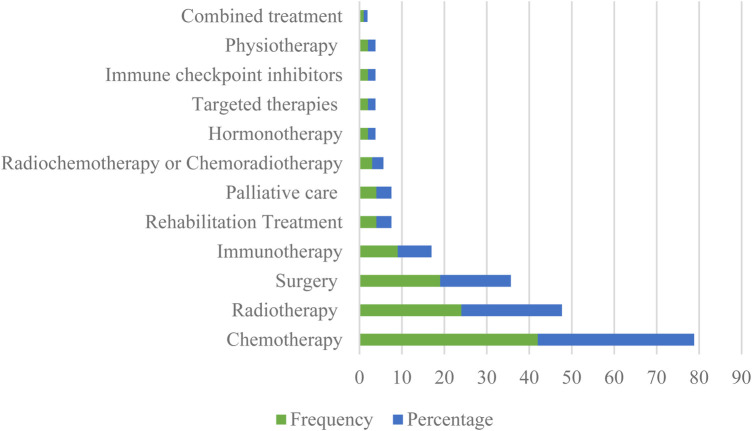
Frequency distribution of studies about ePROM in cancer according to the type of treatments.

The evaluation of ePROM according to the type of cancer treatment is shown in Figure 5. In terms of the types of treatments, 114 treatments had been found and chemotherapy was the most commonly reported treatment, followed by radiotherapy and surgery.

### ePRO questionnaires and measures

**Figure 6:**
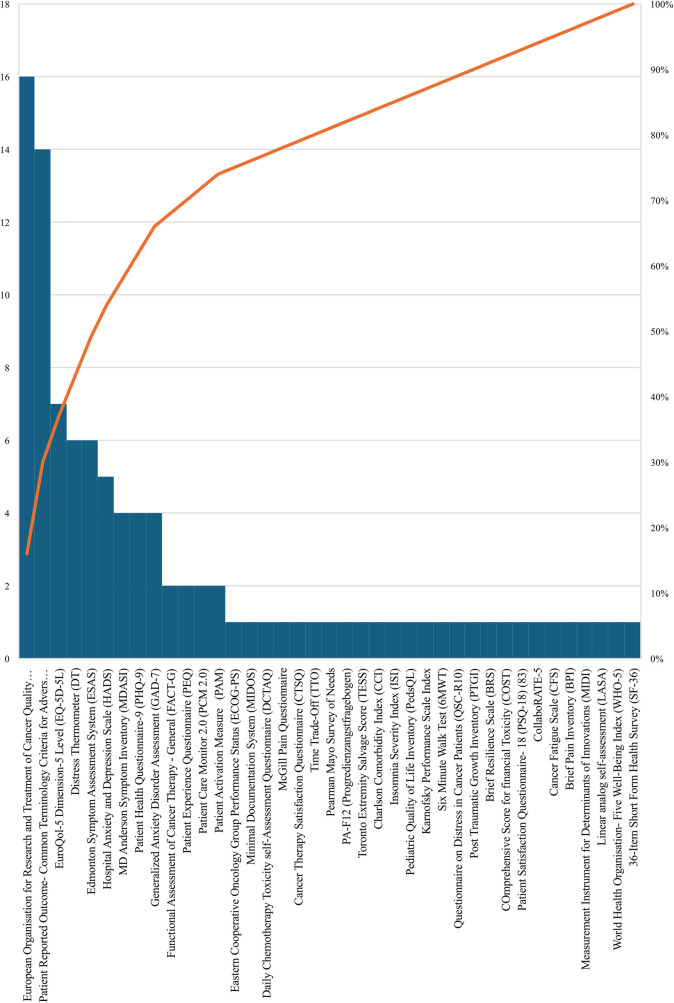
The distribution of ePRO measurements and tools in cancer.

This systematic review identified a diverse range of tools, questionnaires and measurements designed to capture patients’ symptoms and outcomes in ePRO in cancer. According to Figure 6, the most frequently referenced measurements were the European Organisation for Research and Treatment of Cancer Quality of Life Questionnaire Core 30 (EORTC QLQ-C30) contributing valuable insights into the physical, psychological, and social functions of cancer patients (14–29) and the Patient Reported Outcome-Common Terminology Criteria for Adverse Events (PRO-CTCAE) (29–42), providing a comprehensive approach to monitoring symptoms associated with various cancer treatments, each cited in 16 and 14 studies across different cancer types and treatment modalities, respectively.

Among other instruments, the EuroQol-5 Dimension-5 Level (EQ-5D-5l) was cited 7 times, providing significant perspectives on patients’ health status across multiple dimensions (43–49), followed by the Distress Thermometer (DT) (45, 48, 50–53) which provides a succinct yet powerful means for patients to express and quantify emotional distress levels on an 11-point Likert-type scale and the Edmonton Symptom Assessment System (ESAS), tailored for advanced cancer patients to express the intensity of symptoms like pain, fatigue, and anxiety, each referenced 6 times (50–55). Furthermore, the Hospital Anxiety and Depression Scale (HADS) is cited 5 times and categorizing scores into varying levels of anxiety and depression (23, 44, 45, 56, 57), while, the MD Anderson Symptom Inventory (MDASI) is referenced 4 times (44, 45, 47, 58), providing a comprehensive patient-reported outcome measure, investigating into the severity of multiple symptoms impacting cancer patients’ daily lives. Additionally, both the Patient Health Questionnaire-9 (PHQ-9) (11, 45, 55, 59) and the Generalized Anxiety Disorder Assessment (GAD-7) (11, 34, 55, 59) are each mentioned 4 times. Finally, the Functional Assessment of Cancer Therapy - General (FACT-G) (25, 58), the Patient Experience Questionnaire (PEQ) (16, 17), Patient Care Monitor 2.0 (PCM 2.0) (58, 60) and the Patient Activation Measure (PAM) (43, 61) are each referenced 2 times in our study. Table 1 shows a summary of ePRO instruments in oncology and their usage.

**Table 1:** Summary of ePRO instruments in oncology and their usage.

Number	Instrument/Questionnaire	Description
1.	European Organisation for Research and Treatment of Cancer Quality of Life Questionnaire Core 30 (EORTC QLQ-C30) (14–29)	The European Organisation for Research and Treatment of Cancer Quality of Life Questionnaire Core 30 (EORTC QLQ-C30) is a validated instrument designed to measure the physical, psychological, and social functions of cancer patients. It includes multi-item scales and individual items to holistically evaluate patients’ quality of life.
2.	Patient Reported Outcome- Common Terminology Criteria for Adverse Events (PRO- CTCAE) (29–42)	The National Cancer Institute's PRO-CTCAE Measurement System enables cancer patients to self-report symptomatic toxicities during clinical trials. It complements the CTCAE, the standard for adverse event reporting, by incorporating the patient perspective. The system's item library includes 124 items representing 78 toxicities. The PRO-CTCAE questionnaire consists of 41 items covering 22 symptoms commonly associated with cancer treatments, improving the precision of symptom monitoring.
3.	EuroQol-5 Dimension-5 Level (EQ-5D-5l) (43–49)	The EuroQol-5 Dimension-5 Level (EQ-5D-5l) is a comprehensive instrument introduced to enhance sensitivity and reduce ceiling effects. It consists of two parts: a descriptive system with five dimensions and a visual analogue scale (VAS) for self-rated health. Patients provide a nuanced view of their health status across various dimensions.
4.	Distress Thermometer (DT) (45, 48, 50–53)	The Distress Thermometer (DT) is a succinct yet powerful self-assessment tool utilizing an 11-point Likert-type scale represented graphically as a thermometer. Ranging from 0 (no distress) to 10 (extreme distress), patients use the DT to articulate and quantify their emotional distress levels effectively.
5.	Edmonton Symptom Assessment System (ESAS) (50–55)	The Edmonton Symptom Assessment System (ESAS) is a practical self-reporting tool specifically designed for advanced cancer patients. Covering nine common symptoms, it allows patients to express the intensity of symptoms, including pain, fatigue, nausea, and anxiety.
6.	Hospital Anxiety and Depression Scale (HADS) (23, 44, 45, 56, 57)	The Hospital Anxiety and Depression Scale (HADS) is a well-established tool for assessing anxiety and depression levels in cancer patients over the prior week. Comprising two 7-item subscales (HADS-D for depression and HADS-A for anxiety), it categorizes scores into normal, mild, moderate, and severe levels.
7.	MD Anderson Symptom Inventory (MDASI) (44, 45, 47, 58)	The MD Anderson Symptom Inventory (MDASI) serves as a comprehensive patient-reported outcome measure, designed to assess the severity of multiple symptoms experienced by cancer patients. It encompasses physical and psychological symptoms, providing valuable insights into the impact on daily living.
8.	Patient Health Questionnaire-9 (PHQ-9) (11, 45, 55, 59)	The Patient Health Questionnaire-9 (PHQ-9) is a valuable instrument used for diagnosing and monitoring the severity of depression. With nine questions, it includes a specific item screening for suicide ideation, offering a comprehensive view of the patient's mental health.
9.	Generalized Anxiety Disorder Assessment (GAD-7) (11, 34, 55, 59)	The Generalized Anxiety Disorder Assessment (GAD-7) is a concise seven-item instrument designed to measure the severity of generalized anxiety disorder symptoms over the past two weeks. It provides valuable insights into the patient's anxiety levels.
10.	Functional Assessment of Cancer Therapy—General (FACT-G) (25, 58)	The Functional Assessment of Cancer Therapy—General (FACT-G) is a widely used questionnaire for measuring health-related quality of life (HRQOL) in cancer patients. It covers four domains: physical, social/family, emotional, and functional well-being. Version 4 includes 27 items rated on a 0–4 scale, with a total score range of 0 to 108, where higher scores indicate better quality of life.
11.	Patient Experience Questionnaire (PEQ) (16, 17)	The Patient Experience Questionnaire (PEQ) is a comprehensive survey aimed at gathering patient feedback on various aspects of their interaction with healthcare services. It covers communication, accessibility, coordination of care, and overall satisfaction, providing valuable insights for improvement.
12.	Patient Care Monitor 2.0 (PCM 2.0) (58, 60)	The Patient Care Monitor 2.0 (PCM 2.0) is a robust instrument comprising 86 items for women and 80 items for men, rated on an 11-point scale. It covers six subscales, including general physical symptoms, treatment side effects, distress, despair, impaired performance, and impaired ambulation. PCM 2.0 offers a nuanced assessment of patients’ experiences.
13.	Patient Activation Measure (PAM) (43, 61)	The Patient Activation Measure® (PAM) is a versatile survey assessing individuals’ knowledge, skills, and confidence in managing their own health. Available in multiple versions and languages, it provides insights into patients’ ability to take an active role in their health.
14.	Eastern Cooperative Oncology Group Performance Status (ECOG-PS) (44)	The Eastern Cooperative Oncology Group Performance Status (ECOG-PS) is a reliable 6-point scale evaluating functional impairment in cancer patients. It ranges from fully active to restricted in physically strenuous activity, providing valuable insights into patients’ overall well-being.
15.	Minimal Documentation System (MIDOS) (11)	The Minimal Documentation System (MIDOS) is a validated measure for self-assessment of pain and other symptoms in palliative care patients. It allows patients to articulate their experiences, facilitating effective communication with healthcare providers.
16.	Daily Chemotherapy Toxicity self-Assessment Questionnaire (DCTAQ) (36)	The Daily Chemotherapy Toxicity self-Assessment Questionnaire (DCTAQ) is an 11-item self-reported tool specifically developed to assess 10 core chemotherapy-related symptoms. Patients provide information based on symptoms experienced in the past 24 h, offering real-time insights.
17.	McGill Pain Questionnaire (62)	The McGill Pain Questionnaire is a comprehensive tool primarily consisting of three classes of word descriptors—sensory, affective, and evaluative. Patients use these descriptors to specify their subjective pain experience, providing detailed information for effective pain management.
18.	Cancer Therapy Satisfaction Questionnaire (CTSQ) (27)	The Cancer Therapy Satisfaction Questionnaire (CTSQ) is a 21-item instrument assessing various domains related to patient satisfaction with cancer therapy. It covers expectations, feelings about side effects, adherence, convenience, and overall satisfaction, providing a comprehensive view of the patient experience.
19.	Time Trade-Off (TTO) (27)	The Time Trade-Off (TTO) is a choice-based method for eliciting health state utility. It reflects the length of remaining life expectancy a person is willing to trade-off to avoid remaining in a sub-optimal health state, providing valuable insights into patients’ preferences.
20.	Pearman Mayo Survey of Needs (48)	The Pearlman-Mayo Survey of Needs is a comprehensive survey developed to assess various dimensions of cancer patients’ needs. It categorizes needs into physical effects, social issues, psychological aspects, spiritual aspects, and other issues, providing a holistic view for tailored support.
21.	PA-F12 (Progredienzangstfragebogen) (48)	Fear of Progression using the standardized PA-F12 (Progredienzangstfragebogen) questionnaire is a validated measure assessing the fear of disease progression in cancer patients. It covers various aspects of everyday life, offering valuable insights into patients’ emotional well-being.
22.	Toronto Extremity Salvage Score (TESS) (48)	The Toronto Extremity Salvage Score (TESS) is a specialized physical disability measure developed for patients undergoing surgery for extremity tumours. It demonstrates superior measurement properties compared to other scales, offering detailed insights into patients’ physical well-being.
23.	Charlson Comorbidity Index (CCI) (44)	The Charlson Comorbidity Index (CCI) is a widely used tool for classifying comorbid conditions that may influence mortality risk. It provides a comprehensive assessment of comorbidities, aiding in determining survival rates in patients with multiple health conditions.
24.	Insomnia Severity Index (ISI) (45)	The Insomnia Severity Index (ISI) is a seven-item self-report questionnaire assessing the severity of insomnia disorder, its impact on daily life, and treatment response. It provides valuable insights into patients’ sleep-related experiences.
25.	Pediatric Quality of Life Inventory (PedsQL) (63)	The Pediatric Quality of Life Inventory (PedsQL) is a brief measure assessing health-related quality of life in children and young people. It includes proxy reports from parents as well as self-reports from children, offering a comprehensive view of pediatric patients’ well-being.
26.	Karnofsky Performance Scale Index (63)	The Karnofsky Performance Scale Index is a valuable assessment tool for functional impairment in cancer patients. It aids in comparing the effectiveness of different therapies and provides prognostic information based on patients’ performance status.
27.	Six Minute Walk Test (6MWT) (63)	The Six Minute Walk Test (6MWT) is a sub-maximal exercise test designed to assess aerobic capacity and endurance in cancer patients. It provides insights into patients’ physical capabilities and overall fitness.
28.	Questionnaire on Distress in Cancer Patients (QSC-R10) (26)	The Questionnaire on Distress in Cancer Patients (QSC-R10) is a self-reported measure assessing cancer-specific distress. With 10 items covering relevant aspects of everyday life, it offers a nuanced understanding of the psychosocial impact on cancer patients.
29.	Post Traumatic Growth Inventory (PTGI) (57)	The Post Traumatic Growth Inventory (PTGI) is a 21-item scale assessing positive outcomes reported by individuals who have experienced traumatic events. It includes factors such as new possibilities, relating to others, personal strength, spiritual change, and appreciation of life, providing insights into patients’ psychological resilience.
30.	Brief Resilience Scale (BRS) (57)	The Brief Resilience Scale (BRS) is designed to assess the perceived ability to bounce back or recover from stress. A unitary construct of resilience, it includes both positively and negatively worded items, offering a concise yet comprehensive view of patients’ resilience levels.
31.	COmprehensive Score for financial Toxicity (COST) (57)	Financial toxicity, a burden of cancer care itself, is assessed using the COmprehensive Score for financial Toxicity (COST) tool. It is a patient-reported outcome measurement evaluating the financial impact of cancer care on quality of life.
32.	Patient Satisfaction Questionnaire- 18 (PSQ-18) (64)	The Patient Satisfaction Questionnaire- 18 (PSQ-18) is a short-form instrument with 18 items, tapping into seven dimensions of satisfaction with medical care. It covers general satisfaction, technical quality, interpersonal manner, communication, financial aspects, time spent with the doctor, and accessibility and convenience.
33.	CollaboRATE-5 (16)	CollaboRATE-5 is a 5-point Likert scale survey assessing the degree of shared decision-making between patients and healthcare providers. With responses ranging from no effort to every effort, it offers valuable insights into the collaborative nature of healthcare interactions.
34.	Cancer Fatigue Scale (CFS) (55)	The Cancer Fatigue Scale (CFS) is a comprehensive tool assessing cancer-related fatigue. It comprises physical, affective, and cognitive subscales, providing a detailed understanding of the impact of cancer-related fatigue on patients’ lives.
35.	Brief Pain Inventory (BPI) (55)	The Brief Pain Inventory (BPI) rapidly assesses pain severity and its impact on functioning. With a well-defined scale, it categorizes pain into mild, moderate, and severe, offering insights into patients’ pain experiences
36.	Measurement Instrument for Determinants of Innovations (MIDI) (65)	A tool applicable pre- or post-introduction of an innovation, designed to enhance understanding of critical determinants affecting implementation, aiding in targeted innovation strategy.
37.	Linear analog self-assessment (LASA) (39)	Utilized to gauge general well-being and specific factors (mood, pain, nausea, vomiting, appetite, breathlessness, physical activity) in patients undergoing therapy for malignant melanoma, small cell bronchogenic carcinoma (SCBC), or ovarian cancer.
38.	World Health Organisation- Five Well-Being Index (WHO-5) (23)	A brief self-reported measure assessing current mental well-being with five positively worded items, rated on a 6-point Likert scale (0–5).
39.	36-Item Short Form Health Survey (SF-36) (23)	A comprehensive set of generic quality-of-life measures, including eight scales: physical functioning (PF), role physical (RP), bodily pain (BP), general health (GH), vitality (VT), social functioning (SF), role emotional (RE), and mental health (MH).

Table 2 provides a summary of disease-specific ePRO instruments in oncology and their usage. According to Table 2, our systematic review further highlighted the application of disease-specific instruments, Among these, the Quality of Life Questionnaire-Head and Neck 35 (QLQ-H&N35) was referenced in two studies (40, 66), indicating its relevance in assessing health-related quality of life in head-and-neck cancer patients. Similarly, the Quality of Life Questionnaire-Breast 23 (QLQ-BR23) European Organisation for Research appeared in two studies, emphasizing its importance in evaluating various aspects related to breast cancer, such as body image and systemic therapy side effects (16, 17). Another measurement in brain cancer was the EORTC QLQ-BN20 questionnaire (*n* = 1) for assessing the health-related quality of life (HRQoL) in brain cancer patients extracted from EORTIC QLQ to evaluate the quality of life of cancer patients (43). Additionally, the FACT-Melanoma (FACT-M) (49) and Functional Assessment of Cancer Therapy—Ovary (Fact-O) (57) instruments were each cited in one study, showcasing their utility in assessing melanoma and ovarian cancer-specific factors, respectively. Moreover, the FACT-B (Functional Assessment of Cancer Therapy—Breast) (60), European Organisation for Research and Treatment of Cancer Quality of Life Questionnaire Ovarian Cancer 28 (EORTC QLQ-OV28) (61), and European Organisation for Research and Treatment of Cancer Quality of Life Questionnaire Colorectal 29 (EORTC QLQ-CR29) (61) were each referenced once, highlighting their role in evaluating health-related quality of life in breast, ovarian, and colorectal cancer patients, respectively. In terms of prostate cancer, the Expanded Prostate Cancer Index Composite (EPIC) and 8-item Functional Assessment of Cancer Therapy Advanced Prostate Symptom Index (FAPSI) have been used in monitoring prostate cancer (67).

**Table 2:** Summary of disease-specific ePRO instruments in oncology and their usage.

Instrument/questionnaire	Description
Quality of Life Questionnaire-Head and Neck 35 (QLQ-H&N35) (40, 66)	The Quality of Life Questionnaire-Head and Neck 35 (QLQ-H&N35) is a disease-specific module assessing health-related quality of life in head-and-neck cancer patients. With seven multiple-item scales, it covers various aspects such as pain, swallowing ability, and social functioning.
Quality of Life Questionnaire-Breast 23 (QLQ-BR23) (16, 17)	The Quality of Life Questionnaire-Breast 23 (QLQ-BR23) is a specialized module incorporating five multi-item scales to assess various aspects related to breast cancer, including body image, sexual functioning, and systemic therapy side effects.
FACT-Melanoma (FACT-M) (49)	The FACT-Melanoma (FACT-M) is a validated quality-of-life instrument specifically designed for melanoma patients. Developed in 2005, it incorporates the FACT-G along with melanoma-specific items to assess patients’ well-being. FACT-M consists of 51 items, including 27 from the FACT-G subscale, a 16-item Melanoma Subscale (MS), and an 8-item Melanoma Surgery Scale (MSS).
Functional Assessment of Cancer Therapy—Ovary (Fact-O) (57)	The Fact-O (Functional Assessment of Cancer Therapy—Ovary) is a comprehensive instrument combining the FACT-G and an ovarian cancer-specific scale. It provides a detailed assessment of health-related quality of life, considering both general and ovarian cancer-specific factors.
Functional Assessment of Cancer Therapy—Breast (FACT-B) (60)	The FACT-B (Functional Assessment of Cancer Therapy—Breast) is a 37-item instrument measuring multiple domains of health-related quality of life in breast cancer patients. With an emphasis on brevity and patient values, it offers a nuanced understanding of the impact on patients’ well-being.
European Organisation for Research and Treatment of Cancer Quality of Life Questionnaire Ovarian Cancer 28 (EORTC QLQ-OV28) (61)	The European Organisation for Research and Treatment of Cancer Quality of Life Questionnaire Ovarian Cancer 28 (EORTC QLQ-OV28) are specialized instrument for assessing the quality of life and symptoms in ovarian cancer patients. OV28 covers abdominal/GI symptoms.‏
European Organisation for Research and Treatment of Cancer Quality of Life Questionnaire Colorectal 29 (EORTC QLQ-CR29) (61)	The European Organisation for Research and Treatment of Cancer Quality of Life Questionnaire Colorectal 29 (EORTC QLQ-CR29) demonstrates validity and reliability to supplement the QLQ-C30 in assessing patient-reported outcomes during treatment for colorectal cancer. It covers various aspects specific to colorectal cancer treatment.
Expanded Prostate Cancer Index Composite (EPIC) (67)	A comprehensive instrument evaluating patient function and bother post-prostate cancer treatment. Developed with input from a development cohort of localized prostate cancer patients and an expert panel of urological oncologists, radiation oncologists, survey researchers, and prostate cancer nurses.
8-item Functional Assessment of Cancer Therapy Advanced Prostate Symptom Index (FAPSI) (67)	Symptoms/concerns endorsed at a frequency greater than chance probability (17%) were retained for the symptom index and called the FACT Advanced Prostate Symptom Index-8 (FAPSI-8): pain (three items), fatigue, weight loss, urinary difficulties (two items), and concern about the condition becoming worse.
European Organisation for Research and Treatment of Cancer Brain Tumor Questionnaire (EORTC QLQ-BN20) (43)	The European Organization for Research and Treatment of Cancer (EORTC) QLQ-BN20 is a QoL assessment specific to brain neoplasms.

This comprehensive analysis not only shows the prevalence of specific instruments but also indicates the diverse dimensions of patients’ experiences and outcomes that researchers aim to capture in cancer-related studies. The significant use of these tools contributes to a complete understanding of the impact of cancer and its treatment on patients’ well-being, informing tailored interventions and improving the quality of care.

## Discussion

In our study, we conducted a systematic review aimed at synthesizing literature into the landscape of ePROMs and optimising patient-centred oncology care. Additionally, a distribution analysis by year indicates a majority of publications from 2022 to 2023, with the United States as the leading source. Breast cancer emerges as the most frequent cancer, with chemotherapy being the primary treatment. This study explored the electronic Patient Reported Outcomes (ePRO) measurement tools and metrics in cancer care, aiming to provide a comprehensive analysis for future research and clinical practice guidelines.

Our findings emphasize the importance of using a broad set of tools to comprehensively assess the needs and experiences of cancer patients, indicating the necessity of personalizing assessments to accurately record the multidimensional impact of cancer diagnosis and treatment on patients’ quality of life and well-being. This study identifies differences, similarities, and implications for advancing cancer patient-centered care through a comparative analysis with existing studies.

Additionally, a wide range of other instruments were identified, including generic health-related quality of life measures such as the EuroQol-5 Dimension-5 Level (EQ-5D-5l) and disease-specific modules such as the Quality of Life Questionnaire-Head and Neck 35 (QLQ-H&N35) and Quality of Life Questionnaire-Breast 23 (QLQ-BR23). The European Organization for Research and Treatment Quality of Life Test (EORTC QLQ-C30) emerges as the most widely used measure, alongside other significant tools such as PRO-CTCAE, EQ-5D-5l, DT, ESAS, HADS, and MDASI, in evaluating cancer patients. These instruments encompassed domains such as physical functioning, symptoms, psychological distress, and treatment satisfaction, providing a comprehensive evaluation of cancer patients’ experiences. This study is aligned with Samit et al.'s study, which highlights the use of an electronic patient-reported outcome measurement system to improve distress management in oncology (60). Our study explores important tools like EORTC QLQ-C30 and PRO-CTCAE as reference instruments, emphasizing the multidimensional aspects of patients’ outcomes.

Tang et al.'s comparative effectiveness study on patient-reported outcome assessment methods in cancer care complements our work by focusing on improving patient outcomes (45). While our study identifies types of tools and metrics for measuring ePROs in cancer and describes different tools, it contributes to understanding electronic measurement in cancer care. Consistent with Lee et al.'s emphasis on technological approaches like ePRO in enhancing patient participation and treatment monitoring (68) and Wagner et al.'s focus on bringing Patient Reported Outcome Measures to practice for symptom screening in ambulatory cancer care (69), our study indicates the role of technology and the use of ePROs in advancing patient-centered care and symptom monitoring in oncology.

A diverse array of studies further enriches the discourse by exploring varied contexts and interventions related to ePROM implementation. Studies by Patt et al. (70) and Harper et al. (54) delve into the impact of ePROs on adverse events, cost of care, and symptom severity, providing valuable insights into the economic and clinical implications of ePRO integration across different cancer types and treatment modalities. In addition, Gressel et al. utilized the Patient Reported Outcomes Measurement Information System (PROMIS®) to increase referral to ancillary support services for severely symptomatic patients with gynecologic cancer (71). Moreover, investigations into the comparative effectiveness of ePROs against traditional assessment methods, as demonstrated by Warnecke et al. (11) and Moradian et al. (36), elucidate the potential advantages of leveraging technology in symptom management, treatment monitoring, and survivorship care.

In conclusion, our study contributes to a significant understanding of measurement instruments and ePRO measures in cancer care, drawing parallels with existing research to highlight key insights and implications for advancing patient-centered oncology care. By synthesizing diverse perspectives and methodologies, we aim to inform future research, clinical practice, and policy initiatives aimed at optimizing patient outcomes in oncology. Through interdisciplinary collaboration, innovative technology solutions, and patient-centered approaches, we advocate for evidence-based, holistic oncology practice, highlighting the importance of continued research and innovation in leveraging electronic patient-reported outcome (ePRO) measures to enhance cancer care delivery and patient outcomes.

### Limitations

While this study offers valuable insights into electronic patient-reported outcome measures (ePROMs) in cancer care, it is important to acknowledge certain limitations. Firstly, the rapidly evolving nature of healthcare technology raises concerns about the relevance of our findings over time. Additionally, the subjective nature of tool selection across different healthcare settings introduces potential biases in our analysis. The scope of our review may also overlook niche instruments, highlighting the need for further exploration in future studies. Moreover, our survey was limited to published papers from three main databases, suggesting that this study serves as a foundational landscape for prospective research endeavours.

Secondly, while our review included the impact of ePROMs, we did not explore documents related to other technological advancements such as artificial intelligence and wearables. This represents a potential gap in our research that warrants consideration in future investigations. Thirdly, our study excluded other types of papers such as opinion pieces, editorials, and viewpoints, as well as publications in languages other than English. This may have limited the breadth of perspectives included in our analysis. Additionally, we did not solely focus on feasibility studies or patient perspective and experience tests, which could provide valuable insights into the practical implementation and user experience of ePROMs in cancer care. These considerations should be addressed in future research to provide a more comprehensive understanding of the role of technology in improving patient outcomes in oncology.

### Implications

Our study presents several significant implications for both research and clinical practice in oncology. Firstly, the study underscores the pivotal role of electronic patient-reported outcome measures (ePROMs) in improving patient assessment accuracy and advancing patient-centered care delivery within oncology practice. By employing a diverse range of measurement tools tailored to different aspects of cancer care, healthcare providers can gain deeper insights into patients’ needs and experiences, enabling personalized care strategies that address individual patient concerns more effectively.

Moreover, the implications of the study extend beyond clinical practice to research endeavours in oncology. By elucidating the various system tools, questionnaires, and ePRO measurements utilized in cancer patient assessment, the review provides valuable insights into methodological approaches. This knowledge empowers researchers to make informed decisions regarding the selection of appropriate tools tailored to assess specific domains of interest, ultimately contributing to the advancement of knowledge in oncology.

Finally, the findings emphasize the importance of ongoing innovation and refinement in tool development to meet the evolving needs of cancer patients. As the landscape of cancer care continues to evolve, there is a need for continuous improvement and adaptation of ePROMs to ensure their relevance and effectiveness. This underscores the importance of investing in research and development efforts aimed at enhancing the usability, accuracy, and relevance of ePROMs in oncology care.

## Conclusion

In conclusion, our systematic review enhances our understanding of the complex array of system tools, questionnaires, and electronic patient-reported outcome (ePRO) measurements utilized in cancer care. By synthesizing existing literature, we offer valuable insights into the methodologies and technologies shaping oncology research and practice. Moving forward, sustained efforts in tool development, validation, and implementation are crucial for comprehensively assessing and addressing the multifaceted needs of cancer patients, ultimately improving the standard of care and patient outcomes. Our review identifies frequently referenced tools like the EORTC QLQ-C30 and PRO-CTCAE, alongside less commonly utilized instruments, providing a comprehensive overview of available assessment tools. By utilizing a variety of instruments that capture different dimensions of patients’ experiences, clinicians and researchers can enhance their understanding of cancer patients’ quality of life, symptom burden, psychological well-being, and treatment satisfaction. Future research should focus on validating and refining existing instruments while also developing new tools to meet the evolving needs of cancer patients across diverse settings and populations.

## Data Availability

The original contributions presented in the study are included in the article/Supplementary Material, further inquiries can be directed to the corresponding author.

## Appendix

**Table A1:** Search strategy.

Database	Search Strategy
Scopus	TITLE-ABS-KEY (((“electronic patient-reported outcome” OR “electronic patient reported outcome” OR “electronic patient-reported outcome measure” OR “electronic patient reported outcome measure” OR “ePRO” OR “electronic Patient-Self Reporting”) AND (“neoplasms” OR “Oncology” OR “Cancer” OR “tumor”))) AND (LIMIT-TO (DOCTYPE, “re”) OR LIMIT-TO (DOCTYPE, “ar”)) AND [(LIMIT-TO (LANGUAGE, “English”)])
Web of Science	((“electronic patient-reported outcome” OR “electronic patient reported outcome” OR “electronic patient-reported outcome measure” OR “electronic patient reported outcome measure” OR “ePRO” OR “electronic Patient-Self Reporting”) AND (“neoplasms” OR “Oncology” OR “Cancer” OR “tumor”)) (Topic) and Review Article or Article (Document Types) and English (Languages)
PubMed	((“electronic patient-reported outcome"[Title/Abstract] OR “electronic patient reported outcome” OR “electronic patient-reported outcome measure"[Title/Abstract] OR “electronic patient reported outcome measure"[Title/Abstract] OR “ePRO"[Title/Abstract] OR “electronic Patient-Self Reporting"[Title/Abstract]) AND (“neoplasms"[Title/Abstract] OR “Oncology"[Title/Abstract] OR “Cancer"[Title/Abstract] OR “Tumor"[Title/Abstract])) Filter: English

## Appendix

**Table A2:** Summary characteristics of articles included.

Number	Author	Year	Country/State	Objective	Participants	System tools and metrics	Cancer type	Type of treatment
1.	Dickson, et al. (35)	2024	USA	To evaluate the utilization and clinical impact of an electronic patient-reported outcome (ePRO) tool in patients with solid tumours undergoing immuno-oncology (IO) therapy	538 patients in the historical control (HC) cohort, and 1,014 patients in the ePRO cohort, with 319 ePRO users and 695 non-users	Electronic platform for patients to report health status, training for healthcare providers, PRO-Common Terminology Criteria for Adverse Events (PRO-CTCAE) questionnaires, symptom tracking, real-time alerts	Solid tumours [non-small cell lung cancer (NSCLC), melanoma, renal cell carcinoma, bladder cancer, head and neck cancer]	Immuno-oncology therapy
2.	Riedl et al. (14)	2023	Austria	To investigate the ability of adult patients of different age ranges to complete routine ePRO assessments and to identify factors associated with completion and the need for assistance.	5,571 patients (mean age: 60.3 years, range 18 to 93 years) in Inpatient Rehabilitation setting	Electronic assessment of patient-reported outcomes (ePRO), European Organization for the Research and Treatment of Cancer Quality of Life Questionnaire (EORTC QLQ-C30), Hospital Anxiety and Depression Scale	Various cancer types (breast, hemoblastoses, prostate, uterine/ovarian, colon, head/neck, lung, stomach, rectum)	Rehabilitation Treatment
3.	Sprave et al. (64)	2023	Germany	– Investigate the feasibility of integrating electronic patient-reported outcomes (ePROs) in the treatment surveillance pathway for HNC patients during radiotherapy. – Assess the impact of app-based ePRO monitoring on global and disease-specific quality of life and patient satisfaction.	100 enrolled, 93 evaluable	Electronic patient-reported outcomes were collected via a dedicated mobile app (myoncare, Oncare GmbH) using the European Organisation for Research and Treatment of Cancer Core Quality of Life Questionnaire (QLQ-C30), the head and neck cancer module (H&N35), and the validated Patient Satisfaction Questionnaire Short Form (PSQ-18).	Head and neck cancer (HNC) (Oropharynx, Larynx, Hypopharynx, Nasopharynx, and Parotid glands)	Radiotherapy (Definitive radiotherapy, Adjuvant radiotherapy, Reirradiation, and Palliative radiotherapy)
4.	Patt et al. (70)	2023	United States	To evaluate the impact of electronic patient-reported outcomes (ePROs) on adverse events and total cost of care among patients with metastatic cancer enrolled in the Centers for Medicare & Medicaid Services Oncology Care Model (OCM) program	Initially, 1,630 patients with cancer; 831 met the selection criteria, 458 matched patients were identified	Utilized HIPAA-compliant software, Navigating Cancer (NC), Medicare claims data, Adverse Events (AEs), Total Cost of Care Metrics, Outcome Metrics	Metastatic Breast, Chronic leukaemia, Lung, Lymphoma, Multiple myeloma, Prostate, and Small intestine/colorectal cancer	Radiotherapy, and Surgery
5.	Harper, et al. (54)	2023	Canada	1. Describe symptom severity among adolescents and young adults (AYA) with cancer at diagnosis and 1 year after diagnosis. 2. Identify demographic and clinical risk factors for higher symptom severity. 3.. Evaluate symptom trajectories among AYA with cancer during the year following diagnosis. 4. Compare symptom severity and trajectories with older adult patients with cancer.	937 adolescents and young adults; 473 at diagnosis, 322 at 1 year after diagnosis	Edmonton Symptom Assessment System-Revised (ESAS-r) tool measuring:—Pain—Tiredness—Drowsiness—Nausea—Lack of appetite—Shortness of breath—Depression—Anxiety—Overall well-being	Various cancer types, including Breast, Central nervous system, Endocrine, Gastrointestinal, Genitourinary, Gynecologic, Head and neck, Hematologic, Intrathoracic, Melanoma, Sarcoma, etc.	Chemoradiotherapy, Chemotherapy, and Radiotherapy
6.	Oldenburger et al. (72)	2023	Belgium	Explore the opinions of healthcare providers (HCP) active in radiation oncology in Belgium on using ePROMs for symptom follow-up after palliative radiotherapy.	128 respondents, including Radiation Oncologists, Nurses, Radiation Therapy Technologists, Clinical Support Managers, and Quality Managers.	PROMS questions: covering symptoms, side effects of treatment, quality of life,	N/A	Palliative radiotherapy
7.	LA et al. (47)	2023	USA	To rapidly develop, launch through an electronic patient portal, and provide initial validation for a PRO measure of COVID-19 symptom burden in patients with cancer.	600 participants diagnosed with both cancer and COVID-19	MDASI-COVID questionnaire, advanced psychometric validation methods – EQ visual analogue scale (EQ-VAS) – EuroQOL Scales MD Anderson	Various types of cancer in individuals also diagnosed with COVID-19	N/A
8.	Warnecke et al. (11)	2023	Germany	To compare the information provided by ePROMs and nurse-reported assessments to identify overlaps and differences in the assessment of current symptom burden among oncological inpatients.	230 inpatients	Patient Health Questionnaire 8 (PHQ-8), Generalized Anxiety Disorder Scale 7 (GAD-7), Hornheider Screening Instrument (HSI), Minimal Documentation System 2 (MIDOS2)	Soft-tissue sarcoma, Lung, Uveal melanoma, Gastrointestinal, Hepatobiliary and pancreatic cancer	Palliative care
9.	Moradian et al. (36)	2023	Canada	To develop an eHealth platform for cancer patients to manage symptoms and interact with healthcare professionals.	N/A	Common Terminology Criteria for Adverse Events (CTCAE), PRO-CTCAE library, DCTAQ, international guidelines, irAEs observed during clinical trials	Various types of cancer in individuals	Immunotherapy
10.	Mohseni et al. (73)	2023	Iran	Develop a smartphone-based app for electronic reporting of outcomes by patients with prostate cancer	Specialists (*n* = 15), Patients (*n* = 21)	Post-study system usability questionnaire (PSSUQ) Quality of life questionnaire	Prostate cancer	All types of treatment
11.	McMullan et al. (30)	2023	United Kingdom	To assess the usability of the ChemoPRO® app among people with lived experience of cancer.	10 participants with lived experience of cancer.	Included two Patient-Reported Outcome Measures (PROMs): Euroqol EQ5D5l and PRO-CTCAE™.	Leukaemia, Breast, Multiple, Myeloma, Stomach, Bowel, Rectal, Sarcoma	Chemotherapy
12.	Macanovic et al. (31)	2023	Ireland	To investigate the feasibility of implementing a remote patient monitoring system using an electronic patient-reported outcomes (ePROs) platform in a tertiary cancer centre in the Republic of Ireland.	13 patients and 5 staff	Symptom questionnaires through an ePRO mobile phone application (app), NCI-PRO-CTCAE™ assessment scale, patient-facing interface, clinician-facing interface, medication reminders, symptom tracking, communication with the care team	Breast Melanoma Colorectal Lung	Oral Chemotherapy
13.	Holch et al. (25)	2023	United Kingdom	Establish feasibility and acceptability of the eRAPID system	167 (73.2% consented and randomized)	FACT-G (overall and PWB score), EORTC QLQ-C30 summary score, QLQ-C30 global health/QOL score, and EQ5D-VAS	Prostate, lower gastrointestinal, and gynaecological cancers	Chemotherapy
14.	Silvia Hofer et al. (27)	2023	Switzerland	To assess the impact of treatment on health-related quality of life (HRQoL) and patient-reported outcomes in palliative STS treatment.	The study was terminated early due to the COVID-19 pandemic, and only 11 patients were randomized and 10 evaluated.	EORTC QLQ-C30, Cancer Therapy Satisfaction Questionnaire (CTSQ), Time Trade-off (TTO) method.	Soft Tissue Sarcoma (STS)	Chemotherapy
15.	Helissey. et al (38)	2023	Helsinki and France	Effectiveness of electronic patient reporting outcomes, by a digital telemonitoring platform, for prostate cancer care: the Protecty study	61 patients	– The system used a symptomatology questionnaire based on CTCAE v.5.0 to collect patient-reported outcomes (PROs). – An algorithm classified patients into different health states based on reported adverse events.	Prostate cancer	Chemotherapy, Hormonotherapy, Combined treatment
16.	Geese, et al. (48)	2023	Switzerland	Explore the potential of ePROMs in clinical practice for assessing quality of life, functionality, needs, fear of progression, distress, and care quality in sarcoma centres	55 patients from three sarcoma centres	EQ-5D-5l for quality of life, PMSN for unmet needs, NCCN Distress Thermometer (DT) for distress levels, PA-F12 for fear of progression, Toronto extremity salvage score (TESS) for physical functionality	Sarcoma	Surgery, Radiotherapy, Chemotherapy
17.‏	Gvozdanovic et al. (43)	2022	United Kingdom	To assess the feasibility of Vinehealth integration into brain tumour care	Six patients were initially recruited, and four engaged with the Vinehealth application throughout the study period.	Symptom, activity, well-being, medication logs; EORTC QLQ-BN20 (European Organisation for Research and Treatment of Cancer Brain Tumor Questionnaire), EQ-5D-5l (EuroQol 5 dimensions of health questionnaire), and PAM (Patient Activation Measure).	Brain tumours include glioblastoma, metastasis from triple-negative breast carcinoma, and haemangioblastoma.	Surgery, chemoradiotherapy, and radiotherapy
18.‏	Zhang et al. (44)	2022	China	To track patient-reported health status changes over time in Chinese advanced cancer patients and explore the risk factors affecting their health status.	103 patients completed a baseline survey (T = 0) and two follow-up surveys (T1 = 14 days, T2 = 28 days).	EQ-5D-5l instrument (including EQ-VAS) for assessing health status.—MD Anderson Symptom Inventory (MDASI-C) for assessing symptom severity and life interference.—Hospital Anxiety and Depression Scale (HADS) for measuring anxiety and depression symptoms.—Case report form (CRF) for collecting demographic and medical data.—Charlson Comorbidity Index (CCI) for evaluating complications.—Eastern Cooperative Oncology Group Performance Status (ECOG-PS) scale for measuring functional status.	Advanced stages of cancers, including Stage III without curative treatment chance and Stage IV) Lung, gastric, oesophageal, liver, colorectal, and breast cancer	N/A
19.	Zhang et al (74)	2022	China	To compare the efficiency between electronic patient-reported outcomes (ePRO) and traditional follow-up models in cancer immunotherapy.	278 patients (141 in the intervention group, 137 in the control group)	The ePRO follow-up model included: Assessment of immune-related adverse events (irAEs) Quality of life (QOL) assessment using the European Organization for Research and Treatment of Cancer QLQ-C30 Time spent implementing the ePRO model	Gastric, Esophageal, Lung, Pancreatic, Colorectal, Breast, Brain, Liver, Kidney, and others	Patients receiving cancer immunotherapy.
20.	Wickline et al. (75)	2022	United States	Usability and acceptability of the electronic self-assessment and care (eSAC) program in advanced ovarian cancer	Total Sample (*N* = 134); Device Interview Sample (*n* = 18); Usability Interview Sample (*n* = 19) in Ambulatory Setting:	Acceptability E-Scale Score (AES), Usability Interviews, Clinician Surveys, Focus Groups.	Advanced ovarian cancer	Palliative care
21.	Tolstrup et al (49)	2022	Denmark	– To examine the impact of using electronic patient-reported outcomes (ePRO) with triggered alerts as an add-on to standard care on the health-related quality of life (HRQoL) of melanoma patients receiving checkpoint inhibitors. – To investigate the association between immune-related adverse events (irAEs) severity and HRQoL.	Patients (*N* = 138)	EuroQol EQ-5D-5l Index, FACT-M questionnaire	Melanoma	Adjuvant 1st line 2nd line 3rd line, Immune checkpoint inhibitors (CPIs)
22.	Tang et al. (45)	2022	China	To describe the implementation process and evaluation of an ePRO platform for symptom management in cancer patients, share experiences, and assess feasibility, safety, and efficacy.	A total of 161 patients with advanced cancer were enrolled in the study, although completion rates varied across the seven follow-up assessments.	Various validated instruments were used for symptom assessment, including the MD Anderson Symptom Inventory (MDASI), Insomnia Severity Index (ISI), Hospital Anxiety and Depression Scale (HADS), Patient Health Questionnaire-9 Items (PHQ-9), EuroQol 5 Dimensions Questionnaire-5l Version (EQ-5D-5l), and Distress Thermometer. These tools measured a range of symptoms, from pain and fatigue to insomnia and distress.	Patients with advanced cancer, including lung, liver, gastric, oesophagal, colorectal, and breast cancer.	Follow up
23.	Rocque et al. (32)	2022	United States	To adopt a remote symptom monitoring intervention developed in research settings for implementation in real-world clinical settings at two cancer centres.	Phase I: 23 patients; Phase II: 35 patients (Myeloma and Acute Leukemia)	PRO-CTCAE (Common Terminology Criteria for Adverse Events), electronic health data	Lymphoma, Breast, Gastrointestinal, Genitourinary, Myeloma, Acute Leukemia	Chemotherapy, Immunotherapy, or Targeted therapies
24.	Riedl et al. (63)	2022	Austria	To assess the impact of multidisciplinary inpatient rehabilitation on the health-related quality of life (HRQOL) and physical fitness of pediatric cancer survivors.	236 pediatric cancer survivors aged 5-21 years and 478 parents (as proxy respondents).	– Pediatric Quality of Life Inventory (PedsQL) 4.0 Generic Core Scales and PedsQL 3.0 Cancer Module for HRQOL assessment. – Karnofsky Performance Scale Index for functional impairment assessment. – Six-Minute Walk Test (6-MWT) for physical fitness evaluation.	leukemias, lymphomas, Central Nervous System (CNS) tumours Brain, Bone, Soft tissue, Blood and immune system and others.	Rehabilitation Treatment: Set of multidisciplinary therapies including medical and nursing treatment, nutritional counselling, physiotherapy, and psychological therapies
25.	Nordhausen et al. (26)	2022	Germany	To evaluate the implementation of electronic patient-reported outcomes (e-PRO) in inpatient radiation oncology	The study involved a total of 568 patients.	Initial assessment: EORTC QLQ-C30, QSC-R10—Daily symptom monitoring: EORTC single items—Final assessment: EORTC QLQ-C30	Patients with various cancer	Chemotherapy, Radiotherapy
26.	Lee et al. (68)	2022	Korea	To identify factors associated with the adoption and compliance of electronic patient-reported outcome measure (ePROM) among cancer patients in a real-world setting	580 cancer patients	Symptom Assessment, Summary Report, Information for Self-management	Various cancers (e.g., breast, lung, gastric, colorectal, lymphoma, head and neck, others)	Chemotherapy or radiation therapy
27.	Hlubock et al. (57)	2022	United States	To examine the prevalence of psychosocial factors affecting quality of life in ovarian cancer survivors using an electronic patient-reported outcome (ePRO) platform	174 out of 300 ovarian cancer survivors	Hospital Anxiety/Depression Scale (HADS), Post-traumatic Growth Inventory, Brief Resilience Scale, Comprehensive Score for Financial Toxicity (COST), Functional Assessment of Cancer Therapy-Ovarian [FACT-O/health-related quality of life (HRQOL)], Clinical Characteristics	Ovarian cancer	Surgery, Chemotherapy or Radiation therapy
28.	Graf et al. (15)	2022	Germany	To analyze the acceptance and evaluation of a tablet-based ePRO app for breast cancer patients and examine its suitability, effort, and difficulty in the context of HRQoL and sociodemographic factors.	106 women with adjuvant or advanced breast cancer at 2 major university hospitals in Germany.	EORTC QLQ-C30 and FACT-B HRQoL questionnaires, self-reported technical skills, usability evaluation.	Breast Cancer	Adjuvant therapy (Chemotherapy)
29.	Girgis et al. (51)	2022	Australia	To evaluate the processes and success of implementing the PRM system in the routine care of patients diagnosed with lung cancer.	48 patients diagnosed with lung cancer completed 90 assessments during the 5-month implementation period.	The PRM system includes monthly physical and psychosocial well-being assessments using the distress thermometer (Dt) and the Edmonton symptom assessment scale (ESAS). It also includes automated email clinical alerts and tailored patient self-management resources.	Lung cancer	Chemotherapy, Radiotherapy, Surgery, Immunotherapy
30.	Daly et al. (76)	2022	United States	Assess the clinical value of daily electronic patient-reported outcomes (ePROs) for cancer patients undergoing antineoplastic treatment	217 patients (median age 66, 103 women, and 114 men)	ePRO assessments with red and yellow symptom alerts a system-generated alert system with red and yellow alerts triggered by severe or concerning symptoms – Monitoring and recording the fluctuation of symptoms for a week	Breast, head and neck, gastrointestinal, genitourinary, gynaecology, lymphoma, melanoma, thoracic, and soft cancers	Chemotherapy only (includes cytotoxic, antibodies), Immunotherapy only, Combination chemotherapy and immunotherapy, Radiation within 14 d of antineoplastic
31.	Convill et al. (46)	2022	United Kingdom	– Investigate the level of agreement between clinician-reported and self-reported patient smoking status during the first visit to a cancer centre. – Examine the self-reported frequency of smoking cessation after the diagnosis of lung cancer.	195 patients were included in the primary analysis.	– ePROM questionnaires included a question regarding smoking status.—ePROMs included symptom-based questions and Quality of Life (QoL) questions (EQ-5D-5l).	Lung cancer	Palliative, Curative, Neo-adjuvant
32.	Boeke, et al. (77)	2022	Germany	To assess patient acceptance of physical activity (PA) monitoring in an outpatient setting during radiotherapy and to correlate changes in PA with toxicity and changes in quality of life (QoL).	23 patients	Activity trackers, Functional Assessment of Chronic Illness Therapy questionnaire, daily step counts, quality of life measurements	Breast Head and Neck, Lung Anal, Esophageal, and Pancreatic cancer	Radio chemotherapy: Chemotherapy, Radiotherapy
33	Bamgboje-Ayodele, et al. (52)	2022	Australia	To detail the development and implementation of integrated care pathways (ICPs) for electronic collection of patient-reported outcomes (ePROs) in lung cancer patients in oncology settings	96 staff members participated in engagement activities across three hospitals.	Cancer Institute New South Wales (CINSW) Patient Reported Outcome Measures (PRMs) system, Edmonton Symptom Assessment System (ESAS), Distress Thermometer (DT), problem checklist	Lung Cancer	N/A
34.	Takala, et al. (78)	2020	Finland	Assess the usefulness of electronic patient-reported outcomes (ePROs) during adjuvant radiotherapy (RT) in patients with early breast cancer.	253 patients with breast cancer receiving RT	Performance status, anxiety, oedema, skin symptoms, pain in the radiated area, tiredness and fatigue, respiratory symptoms, and other symptoms.	Breast cancer	Radiotherapy Chemotherapy, Surgery, and Hormonal therapy
35.	Strachna (79)	2021	United States	To develop an electronic PROs (ePROs) program for head and neck cancer patients and evaluate its feasibility and impact.	4,154 patients	Patient-reported outcomes measures, electronic survey application (MSK Engage), Tableau Software	Head and neck cancer	Radiotherapy, Surgery, and Chemotherapy
36.	Stormoenet et al (42)	2021	Denmark	To describe Patient-Reported Outcomes (PROs) from patients with metastatic castration-resistant prostate cancer (mCRPC) receiving oncological treatment and compare them with adverse events from registration studies	54 patients with mCRPC receiving medical oncological treatment	The ePRO-CTCAE questionnaire contained 41 items corresponding to 22 symptoms/adverse events associated with the treatment regimens commonly used for mCRPC. Data was stratified by antineoplastic agent administered, and the severity of interference was rated on a scale of 0 to 4.	Metastatic castration-resistant prostate cancer (mCRPC)	Antineoplastic treatment for prostate cancer
37.	Riis et al. (16)	2021	Denmark	To examine the impact on service use, workflow, and workload after introducing ePRO-based individual follow-up for early breast cancer treatment.	Initially, 129 women were assessed, 64 in SFU, and 60 in PIFU; the final assessment included 47 participants for PREMs.	European Organization for Research and Treatment of Cancer Quality of Life Questionnaire Core 30 (EORTC QLQ-C30), EORTC Breast Cancer-Specific Module (QLQ-BR23), CollaboRATE measure, Patient Experience Questionnaire (PEQ), DEFACTUM Patient Involvement Indicator Objectives, Additional electronic Patient-Reported Outcome measures, Various Patient Satisfaction Tools, Additional *ad hoc* Surveys or Questionnaires	Early-stage breast cancer	Surgery, and Chemotherapy in SFU and PIFU,
38.	Ravn, et al. (61)	2021	Denmark	To evaluate the effect of a follow-up supported by electronic patient-reported outcomes (ePRO) on Patient Activation (PA) and Patient Involvement (PI) in patients undergoing intended curative complex surgery for advanced cancer.	187 patients who had undergone intended curative complex surgery for advanced cancer at two different departments at Aarhus University Hospital.	The ePRO included validated questionnaires, including the European Organization for Research and Treatment of Cancer (EORTC) quality of life questionnaire (QlQ) C30, CR29, OV28, and items 6 and 11 from the Hospital and Anxiety Depression scale.	Patients with metastases to the peritoneal surface undergoing intended curative complex surgery for advanced cancer.	Surgery, and systemic chemotherapy
39.	Peltola et al (80)	2021	Finland	To assess the suitability of the Noona ePRO application for patients with cancer, nurses, and doctors at Helsinki University Hospital	– Patients: 44—Health care professionals: 17	– Symptom reporting: Patients used Noona to report symptoms and adverse events.—Questionnaires: Both patients and clinicians answered questionnaires regarding the usability and reliability of Noona.—Usability metrics: This includes assessing ease of use, operability, and learnability of the Noona application.—Reliability: Measured by the subjective opinion of the participants.—Incidence of harmful events: Participants were asked if there were any harmful events related to the use of Noona.	Various solid tumour types	Radiotherapy, Surgery, and Chemotherapy, rehabilitation
40.	Patt et al. (33)	2021	United States	To determine the feasibility of real-world implementation of electronic patient-reported outcomes (ePROs) among patients with cancer at a large community oncology practice.	4,375 patients	The system includes the following tools and metrics:—Patient-Reported Symptoms: Patients were asked to report 14 common cancer-related symptoms each week. – Symptom Questionnaire: The questionnaire is based on the NCI PRO-CTCAE (Patient-Reported Outcomes version of the Common Terminology Criteria for Adverse Events) instrument. – Symptom Reporting via SMS Text, E-mail, or Clinic Collect: Patients had the option to report their symptoms through SMS text messages, e-mail, or by communicating with the clinical team. – Compliance Tracking: The system tracked and monitored patient compliance with symptom reporting over time. It measured the percentage of assessments completed by patients divided by the total number of assessments sent. –Data Visualization: The system provided data visualization of patient-reported symptoms, which were immediately available to a care coordination dashboard. - Risk Stratification: The system stratified patient-reported symptoms by the level of risk, enabling triage nurses and healthcare providers to prioritize and provide immediate care to patients at high risk. –Free-Form Text Box: Patients could add additional toxicities in a free-form text box for reporting.	Breast cancer, Chronic leukaemia, Lung cancer, Lymphoma, Multiple myeloma. And Prostate cancer, Small intestine/colorectal cancer	Radiotherapy, and Surgery
41.	Licht et al. (56)	2021	Austria	Investigate cancer survivors’ health-related quality of life (HRQOL), specific deficiencies related to underlying disease or treatment, and benefits of rehabilitation in a variety of cancer entities.	4,401 cancer survivors	– EORTC-QLQ-C30 questionnaire for HRQOL, symptoms, and functions assessment. Hospital Anxiety and Depression Scale (HADS) for psychological distress evaluation.	Various cancer entities, including head and neck, esophageal, gastric, colon, rectal, liver, pancreatic, lung, skin, breast, uterine, ovarian, prostate, testicular, renal, bladder, brain, thyroid, malignant lymphomas, multiple myeloma, leukaemias, and other cancer types	Rehabilitation program:Guidance and treatment by a physician Nursing procedures Psychooncology, including individual counselling and biofeedback Group psycho-oncological counselling Psychological counselling, including sexual therapy Psychoeducative lectures Educational presentations focusing on motivation and lifestyle modification Cognitive and perception training Creative therapies Social counseling Speech therapy Nutritional advice Individual and group occupational therapy Physiotherapy, both individual and group sessions Medical training therapy, including aerobic and resistance training Remedial massages Manual lymphatic drainage Hydrogymnastics Electrotherapy Therapeutic ultrasound Thermotherapy Inhalation therapies
42.	Lee et al. (59)	2021	South Korea	To evaluate the degree of depression and anxiety in patients with breast cancer during the treatment period and short-term follow-up.	137 patients with breast cancer	PHQ-9 and GAD-7 for depression and anxiety assessment	Breast cancer	Chemotherapy or radiation and at 6-month follow-up examination after the Surgery
43.	Lapen et al. (34)	2021	United States	Develop and study the implementation of a remote system for toxicity assessment and management of acute breast radiation side effects using electronic patient-reported outcomes (ePROs)	678 patients	- Patient-Reported Outcomes Common Terminology Criteria for Adverse Events (PRO-CTCAE)—Generalized Anxiety Disorder 2-item (GAD-2) screening tool—Likert-type scale questions for frequency, severity, and distress related to symptoms—Questions about anxiety using the GAD-2	Breast cancer	Radiotherapy, and Chemotherapy
44.	Judge et al. (62)	2021	United States	To identify implementation issues and evaluate the efficacy of an electronic patient self-reporting pain device in community-based cancer clinics.	178 cancer patients (33 in the pilot phase and 145 in the RCT phase) in community-based clinics	PAINReportIt® is an electronic version of the McGill Pain Questionnaire, which measures pain parameters including location, intensity, quality, and pattern.	Various types of Cancer patients	Radiotherapy
45.	Generalova et al. (81)	2021	United States	Feasibility, implementation, and healthcare utilization outcomes of an electronic PRO (ePRO) application for cancer patients at an academic medical centre.	72 patients	Patient feasibility (greater than 70% completion of questionnaires), patient acceptability (neutral or higher satisfaction), symptom responses, ambulatory healthcare utilization	Patients with advanced cancer in the thoracic, gastrointestinal, and genitourinary oncology groups	Medicine treatment, chemotherapy, or immunotherapy
46.	Doolin et al. (82)	2021	United States	To assess the feasibility of implementing an electronic patient-reported outcomes (ePRO) system for patients starting oral chemotherapy at a cancer centre improving patient monitoring, and symptom assessment.	62 patients who started a new oral chemotherapy regimen agreed to receive online ePROs, and 25 of them completed the ePRO (40% completion rate). A historical cohort of 50 patients was also used for comparison.	An ePRO questionnaire was designed in REDCap and included questions related to the treatment start date, administration issues, new or concerning symptoms, and financial toxicity concerns. Responses triggered clinical outreach. The primary outcome was time to first symptom assessment, along with other outcome measures such as time to first clinical action and ED visits/hospitalizations.	N.A	Oral Chemotherapy
47.	Absolom et al. (83)	2021	United Kingdom	To evaluate the impact of eRAPID on symptom control, healthcare use, patient self-efficacy, and quality of life in a patient population predominantly treated with curative intent during chemotherapy	508 consenting patients and 55 health professionals	Online symptom self-reporting, automated algorithm-driven severity-dependent patient advice, and immediate integration of the self-reports in electronic patient records (EPRs).	Colorectal, breast, or gynaecological cancers	Chemotherapy
48.	Zylla et al. (84)	2020	United States	To assess the feasibility of using electronic patient-reported outcomes (ePROs) for symptom monitoring in patients with advanced cancer	80 patients with stage IV non-hematologic malignancies on chemotherapy	The study used the Patient-Reported Symptom Monitoring (PRSM) survey, which is a 23-question ePRO tool for oncology patients. Symptom responses were categorized on a scale from 0 (none) to 4 (very severe). Severe symptoms were highlighted if the pain rating was ≥7 or any other symptom rated ≥3 (severe or very severe).	Stage IV non-hematologic malignancies include lung, colorectal, prostate, pancreas, head and neck, oesophagal and stomach, breast, ovarian, cervical, endometrial, liver/bile duct, and kidney cancers.	Chemotherapy
49.	Tran et al. (67)	2020	United States	To explore the feasibility and acceptability of collecting electronic patient-reported outcomes (ePROs) using validated health-related quality of life (HRQoL) questionnaires for prostate cancer.	29 patients in total; 1 patient excluded from analysis	Apple ResearchKit software, 26-item Expanded Prostate Cancer Index Composite (EPIC), 8-item Functional Assessment of Cancer Therapy Advanced Prostate Symptom Index, qualitative interviews	Prostate cancer	N/A
50.	Tolstrup et al. (41)	2020	Denmark	Assess the electronic tool's impact on reducing severe adverse events by 50% in melanoma patients undergoing immunotherapy.	146 melanoma patients participated in the study.	Patients in the intervention group received a tablet computer with a SIM card to ensure participation in the web-based evaluation. The PRO-CTCAE item library was used for patient reporting. Weekly reporting was chosen since it is the preferred recall period in PRO-CTCAE questionnaires.	Metastatic melanoma patients receiving immunotherapy.	Immunotherapy
51.	Sandhu et al. (85)	2020	United States	To gain insights into oncologists’ perspectives regarding the incorporation of electronic patient-reported outcomes (ePROs) into routine cancer care at an academic centre	16 oncologists with diverse subspecialties and experience in various cancer types	– Customizable ePRO questionnaires—Data visualization tools—Ease of use—Documentation features, including dot phrases for efficient data entry	Genitourinary, Breast, GI, Sarcoma, Urologic, Thoracic	Palliative medicine
52.	Riis et al. (17)	2020	Denmark	Evaluate patients’ satisfaction with care provided using electronic patient-reported outcomes (ePROs) to individualize follow-up care for women with early breast cancer receiving adjuvant endocrine therapy	134 women	ePROs, Patient Experience Questionnaire (PEQ), EORTC QLQ-C30, EORTC breast cancer module (QLQ-BR23)	Early breast cancer	Surgery, and Adjuvant endocrine therapy
53.	Richards et al. (86)	2020	United Kingdom	To evaluate the feasibility of a real-time electronic symptom monitoring system for patients after discharge following cancer-related upper gastrointestinal surgery	40 participants in the study	– Online symptom-report questionnaire—Clinically derived algorithms for symptom severity—Integration with EHR (Electronic Health Records)	Cancer-related upper gastrointestinal surgery (oesophageal, gastric, hepato-pancreato biliary cancer)	Surgery, Chemotherapy
54.	Mowlem et al. (87)	2020	United Kingdom	To understand the impact of anticancer treatment on oncology patients’ ability to use electronic solutions for completing patient-reported outcomes (ePRO).	Seven individuals with a cancer diagnosis and treatment experience.	The researchers assessed the usability of the eCOA software solutions, including electronic diaries, questionnaires, and response scale types, on both tablet and mobile devices.	Breast, Prostate, and Colon/bowel	Chemotherapy
55.	Karamanidoua et al. (88)	2020	Greece	To develop a novel ePRO-based palliative care intervention for cancer patients by eliciting end-users needs and judgments of the MyPal system and recommendations for improvement.	Nine patients with Chronic Lymphocytic Leukemia (CLL)	MyPal uses eHealth technologies to support cancer patients and healthcare professionals. It includes electronic Patient Reported Outcome (ePRO) assessments, smartphone applications, smart wristbands, and personalized medical information searches. Metrics and measures include symptom reporting, information access, personalized data, and more.	Chronic Lymphocytic Leukemia (CLL) and Myelodysplastic Syndromes (MDS)	Palliative Care
56.	Howell et al. (55)	2020	Canada	To implement electronic Patient Reported Outcomes (e-PROs) in ‘real-world’ oncology practices for personalized management of generic and targeted symptoms of pain, fatigue, and emotional distress (depression, anxiety).	Over 6000 patients completed e-PROs	– ESAS-r (Edmonton Symptom Assessment System revised version)—BPI (Brief Pain Inventory)—CFS (Cancer Fatigue Scale)—PHQ-9 (Prime Health Questionnaire)—GAD-7 (Generalized Anxiety Disorder)—Other tools for symptom management and assessment	Lung and sarcoma cancer	chemotherapy, Radiotherapy, Supportive and palliative care
57.	Girgis et al. (53)	2020	Australia	Evaluate the effectiveness of the PROMPT-Care web-based system in a diverse population of cancer patients by reducing emergency department presentations and other health service outcomes.	328 patients received the intervention, and 1312 patients were matched as controls.	Electronic PRO physical symptom and psychosocial well-being assessments, automated electronic clinical alerts, and online patient self-management resources. Distress Thermometer (DT), Edmonton Symptom Assessment Scale (ESAS), and Supportive Care Needs Survey-Screening Tool 9 were used for assessments. Clinical feedback reports provided recommended clinical actions and referrals. Clinical alerts were generated when individual ePRO item scores breached predefined thresholds on two consecutive assessments. Patient self-management resources included domain-specific webpages and information resources.	Patients with solid tumors	Chemotherapy, Radiotherapy, Surgery, and Immunotherapy
58.	Dronkers et al. (89)	2020	Netherlands	To evaluate the implementation of an electronic patient-reported outcome measures (ePROs) system, in the routine care of head and neck cancer (HNC) patients.	-Quantitative: HM group (45 patients), Standard care group (46 patients)—Qualitative: Interviews with 15 HM patients	- Internationally validated questionnaires measuring physical problems, psychosocial symptoms, and health-related quality of life (HRQoL) of HNC patients.—Results are graphically displayed in the electronic health record (EHR) and used for monitoring and feedback to patients.	Head and neck cancer	N/A
59.	Biran et al. (90)	2020	United States	Evaluate the acceptability and appropriateness of an electronic patient-reported outcome (ePRO) intervention for patients with relapsed and refractory multiple myeloma (RRMM) and explore its impact on clinic workflow	11 patients with RRMM were recruited, and 9 patients completed the study	The app facilitated the reporting of 17 common RRMM Patient-Reported Outcomes (PROs), including severity, frequency, and interference measures. The data generated alerts for the clinic and provided self-management guidance to patients.	Relapsed and Refractory Multiple Myeloma (RRMM)	N/A
60.	Warringtonet al. (91)	2019	United Kingdom	To field test the eRAPID system, an online tool for monitoring and managing adverse events in patients with cancer during treatment.	12 patients receiving chemotherapy for early breast cancer and 10 health professionals (oncologists and specialist nurses).	The eRAPID system allows patients to complete weekly online symptom reports, provides severity-based self-management advice, and sends notifications to contact the hospital for severe symptoms. Patient data is available in electronic records for staff to review. Metrics include the frequency of online symptom report completion and severe symptom notifications.	Patients with early breast cancer were tested in the field usability study. The eRAPID system is being evaluated in a larger population, including patients with breast, gynaecology, or colorectal cancer.	Chemotherapy
61.	Krogstad et al. (92)	2019	Norway	To evaluate the usability of the EirV3 system used for patient-reported outcome measures (PROMs) in cancer care	37 patients, 17 physicians	EirV3 includes questions assessing 19 common cancer-related symptoms and additional questions on functioning and nutritional status. It presents graphical representations of symptom intensity over time and patient answers to follow-up questions.	Breast, Gastrointestinal, Lymphomas, Prostate, Gynecological, Lung, Malignant melanoma, and Testicular cancers	Palliative, Chemotherapy, and Radiotherapy
62.	Kikawa et al. (18)	2019	Japan	Evaluation of health-related quality of life (HRQOL) monitoring from home among metastatic breast cancer (MBC) patients using the Computer-Based Health Evaluation System (CHES)	16 MBC patients who received outpatient chemotherapy or endocrine therapy, both with and without targeted therapy.	CHES is a platform that electronically collects patient questionnaires developed by the EORTC QOL group. The system uses a Japanese version of the EORTC QLQ C30 questionnaire for HRQOL evaluation.	Metastatic breast cancer (MBC)	Outpatient chemotherapy or endocrine therapy both with and without targeted therapy.
63.	Iivanainen et al. (37)	2019	Finland	Investigate whether symptoms collected by the Kaiku Health ePRO tool on cancer patients receiving immune checkpoint inhibitors (ICI)	37 patients	– PRO-CTCAE and 18 adaptive questions assessing the presence and severity of symptoms.—QLQ-C30 questionnaires used for quality of life (QoL).	Various types of cancer	Immune checkpoint inhibitor (ICI) therapy
64.	Gressel et al. (71)	2019	United States	To establish feasibility and acceptability of PROMIS ePRO integration in a gynecologic oncology outpatient clinic and assess if it can help identify severely symptomatic patients and increase referral to supportive services.	336 patients in the Gynecologic Oncology Clinic:	– Physical Function—Pain Interference—Fatigue—Depression—Anxiety—Sexual Function (Lubrication, Global Sexual Satisfaction, Interest in Sexual Activity, Vaginal Discomfort)	Gynecologic cancer	– Post-operative – Chemotherapy – Radiotherapy
65.	Brant et al. (50)	2019	United States	To determine the perception of patients and providers from patient-reported outcomes	121 women (51 with gynecologic cancer and 70 with breast cancer)	– Edmonton Symptom Assessment Scale—National Comprehensive Cancer Network's (NCCN's) Distress Thermometer (DT)- Other symptom and quality-of-life surveys—Patient assessment tools—Published guidelines (NCCN, Oncology Nursing Society)	Breast Cancer, Gynecologic Cancer	N/A
66.	Kerry N. L. Avery, et al. (93)	2019	United Kingdom	To develop a hospital EHR-integrated ePRO system to improve the detection and management of complications post-discharge following cancer-related surgery	Phase 1: 18 patients, Phase 2: 59 participants who provided 444 complete self-reports	The ePRO system employed 35 symptom-report items from validated European Organisation for Research and Treatment of Cancer (EORTC) questionnaires. Clinical algorithms were created for symptom severity-dependent patient advice and clinician alerts. Self-management advice was informed by clinician-patient consultations, patient interviews, and a review of hospital patient information leaflets and patient support websites.	Cancer-related major abdominal surgery	Surgery
67.	Schepers, et al. (65)	2016	Netherlands	Determine the fidelity of the KLIK method as implemented in outpatient pediatric cancer care	205 children with newly diagnosed cancer	– Online tool—Health-related quality of life (HRQoL) questionnaires—Electronic patient-reported outcomes (ePROs)—Measurement Instrument for Determinants of Innovations (MIDI)	Pediatric cancer: Leukemias/lymphomas, Solid Tumors, and Brain Tumors	N/A
68.	Niska et al. (39)	2017	United States	To assess changes in quality of life (QOL) and adverse events (AEs) during radiotherapy (RT) for head-and-neck cancer using electronic patient-reported QOL (PROQOL) data.	65 patients	– Linear analog self-assessment (LASA) was used to measure 12 health-related QOL domains.—LASA domains included overall QOL, mental well-being, physical well-being, emotional well-being, social activity, spiritual well-being, pain frequency, pain severity, fatigue level, level of support, financial concerns, and legal concerns.—Adverse events were graded using the Common Terminology Criteria for Adverse Events (CTCAE).—Clinical characteristics, tumour characteristics, and supportive care interventions were recorded.	Head and neck cancer	Radiotherapy
69.	Lucas et al. (94)	2017	United States	To report on the establishment of a unified, electronic PRO infrastructure and	773 eligible patients, with 688 (89%) enrolled preoperatively	Validated 21-item web-based questionnaire for urinary function, erection function, and sexual interest and satisfaction. Additional questions related to quality of life, relationship status, and use of erectile aids.	Prostate cancer	Surgery
70.	Holch et al. (95)	2017	United Kingdom	To develop a system for patients to self-report and manage adverse events (AE) during and after cancer treatment	Patient advocates (*N* = 9), patients (*N* = 13), and staff (*N* = 19) participated in usability testing	– ePRO data collection interface (QTool)—Real-time integration of PRO data with EPR (QStore)—Hierarchical algorithms for AE grading—Immediate, automated advice for managing AE—Email notifications for severe AE—Secure data transfer through NHS network	Breast, gynaecological, colorectal, pelvic radiotherapy, upper gastrointestinal surgery	Surgery, Radiotherapy
71.	Hartkopf et al. (19)	2017	Germany	To investigate the willingness, and assess specific needs, and barriers of adjuvant breast cancer (aBC) and metastatic breast cancer (mBC) patients in nonexposed and exposed settings before implementing digital electronic Patient-Reported Outcome (ePRO)	202 participants (nonexposed group: 96, exposed group: 106)	Socioeconomic variables, Health-Related Quality of Life (HRQoL) according to EORTC QLQ-C30, preexisting technical skills, the general attitude toward electronic-based surveys, and potential barriers about health status.	Breast cancer	Chemotherapy
72.	Absolom et al. (96)	2017	United Kingdom	To improve the safe delivery of cancer treatments, enhance patient care, and standardize adverse event (AE) documentation	Internal pilot phase with 87 participants, full trial target sample of 504 participants	– Patients can log in to QTool to access the eRAPID symptom questionnaire—Provides patient advice on managing mild AEs—Alerts for severe AEs sent to clinical teams via email—Integration with electronic patient records (EPR) for access to AE reports—Usability testing with 14 breast cancer patients—System Usability Scale (SUS) for participant feedback	Breast, colorectal and gynaecological cancer	Chemotherapy
73.	Schougaard et al. (23)	2016	Denmark	To implement telepatient-reported outcomes (telePRO) as the basis for follow-up in chronic and malignant diseases using the generic PRO system AmbuFlex	AmbuFlex was implemented in nine diagnostic groups in Denmark. A total of 13,135 outpatients from 15 clinics have been individually referred. -Response rates for the initial questionnaire ranged from 81% to 98% in different patient groups	WHO-5, SF-36, HADS, EORTC QLQ-C30. *ad hoc* items were developed in five projects PRO data collection, PRO-based automated decision algorithm, PRO-based graphical overview for clinical decision support	Prostate, colorectal cancer	Chemotherapy, and Radiotherapy
74.	Peltola et al. (40)	2016	Finland	Assess the suitability of Kaiku® (an ePRO application) for collecting patient-reported outcomes (PROs) related to early side effects of radiotherapy and health-related quality of life in head and neck cancer (HNC) patients.	Nine HNC patients were approached, and five consented to participate.	– Patients used Kaiku® for self-assessment of side effects related to treatment on a scale adapted from CTCAE v. 4.03.—Quality of life was monitored using the Finnish versions of the 15D and the EORTC QLQ-H&N35 instruments.—Communication in free-text format was allowed.	Head and neck cancer (HNC)	Radiotherapy
75.	Mayrbäurl et al. (24)	2016	Austria	To assess health-related quality of life (HRQOL) in patients with advanced colorectal cancer across different lines of palliative chemotherapy	100 consecutive patients with colorectal carcinoma	– HRQOL data was collected using the EORTC QLQ-C30 questionnaire, which includes multiple domains such as physical functioning, emotional functioning, pain, fatigue, dyspnea, appetite, and more.	Colorectal cancer	Palliative chemotherapy
76.	Graf et al. (20)	2016	Germany	To determine the extent to which existing computer skills, disease status, health-related quality of life, and sociodemographic factors affect patients’ willingness to use electronic methods of data collection (ePRO)	96	EORTC QLQ-C30, EQ VAS, technology skills, willingness to use electronic PRO surveys	Breast cancer	N/A
77.	Duregger et al. (97)	2016	Austria	To develop a concept and implement a prototype for introducing electronic Patient Reported Outcomes (ePRO) into the existing neuroblastoma research network by applying Near Field Communication (NFC) and mobile technology.	N/A	– NFC technology for contactless data transmission—Quick Response (QR) Codes for data capture—Apache Cordova for cross-platform application development—OpenClinica for data capture via Simple Object Access Protocol (SOAP)—Patient ID cards for secure patient authentication and linking	Neuroblastoma (the primary focus of the study), but the system's applicability extends to other pediatric cancers and rare diseases.	N/A
78.	Cowan et al. (29)	2016	United States	The assessment, and feasibility of acceptability and satisfaction of a Web-based system for capturing patient-reported outcomes (PROs) in the immediate postoperative period in gynecologic cancer surgery patients.	96 eligible patients	The system included surveys based on the patient adaptation of the NCI Common Terminology Criteria for Adverse Events (CTCAE) version 3.0 and the European Organization for Research and Treatment of Cancer (EORTC) QLQ-C30 version 3.0. Alerts were generated based on patient-reported symptoms.	Gynecologic cancer	Surgery
79.	Wintner et al. (21)	2015	Austria	Assessment, and the feasibility of routine clinic-ePRO/home-ePRO with the Computer-based Health Evaluation System (CHES) software.	– 113 patients for clinic-ePRO—45 patients for home-ePRO	– Electronic PRO (ePRO) assessment—Assessment tools: European Organization for Research and Treatment of Cancer Quality of Life Questionnaire (EORTC QLQ-30) and EORTC QLQ-C30—iOS app for iPad2—Web-based assessment	Gastrointestinal, glioma, gynaecological, lung, neuroendocrine, and testicular cancers	Follow-up, and chemotherapy
80.	Wagner et al. (69)	2015	United States	To integrate electronic patient-reported outcome (ePRO) assessment into the electronic health record (EHR) and clinical workflow for symptom screening in ambulatory cancer care.	636 women receiving gynecologic oncology outpatient care	PROMIS CATs for fatigue, pain interference, physical function, depression, and anxiety. Checklists for psychosocial concerns, information needs, and nutritional concerns. PROMIS T-scores with severity thresholds.	Ovarian, Uterine, Cervical, Other female genital malignancy	Gynecologic oncology outpatient care
81.	Erqi L. Pollom, et al. (66)	2015	United States	To evaluate the feasibility of eQOL data collection using a touch-screen tablet device in patients undergoing treatment for head and neck cancer	50 patients	– Surveys: EORTC-QLQ-C30 and EORTC-QLQ-H&N35 administered on a touch-screen tablet device (iPadTM).—Data capture and analysis: Qualtrics, providing automated export to common data analysis packages such as Excel, SAS, Stat, R, and SPSS. Qualtrics also allows the de-identification of data in the export file.	Head and neck cancers	Radiotherapy, and chemotherapy
82.	Smith et al. (60)	2014	United States	Demonstrate how an electronic patient-reported outcome (ePRO) system can aid in distress management in oncology.	17,338	– Review of Systems instrument Patient Care Monitor (PCM)- v2.0 Distress and Despair subscales—FACT-B (Quality of Life)	Breast, lung, and gastrointestinal cancer patients	N/A
83.	Wintner et al. (22)	2013	Austria	To assess the quality of life (QOL) of lung cancer patients undergoing chemotherapy (CT) across multiple treatment lines.	187	The EORTC QLQ-C30 questionnaire was used, which includes Functioning Scales (Physical, Social, Role, Cognitive, and Emotional Functioning), a Global QOL scale, and Symptom Scales (Fatigue, Pain, Dyspnoea, Appetite Loss, Sleep Disturbance, Constipation, Diarrhoea, Financial Difficulties), and additional items for taste alterations.	Lung cancer	Chemotherapy
84.	Zabernigg et al. (28)	2012	Austria	To investigate QOL trajectories from adjuvant treatment to palliative 3rd-line therapy	80 patients (Pancreatic cancer and cancer of the bile ducts)	EORTC QLQ-C30 questionnaire, Computer-based Health Evaluation System (CHES)	Pancreatic cancer and Bile duct Cancer	Chemotherapy
85.	Abernethy et al. (58)	2010	United States	Demonstrate a rapid learning healthcare model in an academic oncology clinic using electronic patient-reported outcomes (ePROs) as foundational data	Metastatic breast cancer (*n* = 65) and gastrointestinal cancer (*n* = 113) patients in Duke Cancer Clinics	e/Tablets, Patient Care Monitor (PCM), FACT-G, MD Anderson Symptom Inventory (MDASI), NCCN Distress Scale	Breast and gastrointestinal cancer	N/A
